# Singularity of the Spectrum for Smooth Area-Preserving Flows in Genus Two and Translation Surfaces Well Approximated by Cylinders

**DOI:** 10.1007/s00220-020-03895-x

**Published:** 2020-12-08

**Authors:** Jon Chaika, Krzysztof Frączek, Adam Kanigowski, Corinna Ulcigrai

**Affiliations:** 1 Department of Mathematics, University of Utah, 155 South 1400 East, Salt Lake City, Utah 84112 USA; 2 Faculty of Mathematics and Computer Science, Nicolaus Copernicus University, ul. Chopina 12/18, 87-100 Toruń, Poland; 3 Department of Mathematics, University of Maryland, 4176 Campus Drive, William E. Kirwan Hall, College Park, MD 20742-4015 USA; 4 Institut für Mathematik, Universität Zürich, Winterthurerstrasse 190, 8057 Zurich, Switzerland

## Abstract

We consider smooth flows preserving a smooth invariant measure, or, equivalently, locally Hamiltonian flows on compact orientable surfaces and show that, when the genus of the surface is two, almost every such locally Hamiltonian flow with two non-degenerate isomorphic saddles has singular spectrum. More in general, singularity of the spectrum holds for special flows over a full measure set of interval exchange transformations with a hyperelliptic permutation (of any number of exchanged intervals), under a roof with symmetric logarithmic singularities. The result is proved using a criterion for singularity based on tightness of Birkhoff sums with exponential tails decay. A key ingredient in the proof, which is of independent interest, is a result on translation surfaces well approximated by single cylinders. We show that for almost every translation surface in any connected component of any stratum there exists a full measure set of directions which can be well approximated by a single cylinder of area arbitrarily close to one. The result, in the special case of the stratum $\mathcal {H}(1,1)$, yields rigidity sets needed for the singularity result.

This paper provides a first general result on the nature of the spectrum for typical smooth area-preserving flows on surfaces of higher genus. Area-preserving flows are one of the most basic examples of dynamical systems, studied since Poincaré at the dawn of the study of dynamical systems. We consider the natural class of smooth flows preserving a smooth invariant measure on surfaces of genus $g\ge 1$, also known as *locally Hamiltonian flows* (see Sect. 2.1) or equivalently multivalued Hamiltonian flows. The study of locally Hamiltonian flows has been pushed since the 1990*s* by Novikov and his school for its connection with solid state physics and pseudo-periodic topology (see e.g. [Nov] and [Zor]). Locally Hamiltonian flows arise indeed in the Novikov model of motion of an electron in a metal under a magnetic field - in this semi-classical approximation, the (compact) surface which constrains the motion is then the (quotient of the) periodic Fermi energy level surface of the metal. Basic ergodic properties (such as minimality and ergodicity) of such flows can be deduced^1^ from classical results (such as [Kea, Mas82, Ve]) on translation flows (which are well understood thanks to the connection with Teichmüller dynamics, see e.g. [AF, Mas06]). On the other hand, finer ergodic and spectral properties depend on the nature of the locally Hamiltonian parametrization and on the type of fixed points of the flow.

In the past decades, there have been many advances in our understanding of finer ergodic properties of locally Hamiltonian flows, in particular mixing and rigidity properties, starting from a conjecture by Arnold on mixing in locally Hamiltonian flows in genus one (see [Arn] and [KS]), which led naturally to the study of mixing (and weak mixing) in higher genus locally Hamiltonian flows [Ul07, Ul09, Sc, Ul11, Rav], up to recent results on mixing of all orders [FK, KKU] and disjointness phenomena [KLU, BK], some of which were achieved adapting to the world of smooth flows with singularities tools inspired from homogeneous dynamics and the work of Marina Ratner (a quick review of the known result is presented in Sect. 2.8).

The spectral properties (and in particular what is the spectral type, see Sect. 3.1 for definitions) of locally Hamiltonian flows is a natural question, which has been lingering for decades (see e.g. [KT, Section 6] and [L]).^2^ Results on the spectrum of the operator, though, are very rare. In an early work by Frączek and Lemańczyk [FL03], spectral properties of special flows over rotations with single symmetric logarithmic singularity (see Sect. 2.8) are examined. In [FL03, Theorem 12] it is shown that (for a full measure set of rotation numbers) such special flows have purely singular continuous spectrum.^3^ This gives examples of locally Hamiltonian flows on surfaces of any genus $\ge 1$ with singular continuous spectrum (see [FL03, Theorem 1]). This result shows that, when one can prove absence of mixing and some form of (partial) rigidity, it might be possible to deduce singularity of the spectrum. A recent spectral breakthrough, which goes in the opposite direction, was achieved by Fayad, Forni and Kanigowski in [FFK], who showed that a class of smooth flows on surfaces of genus one (which can also be represented as special flows over rotations, see Sect. 2.8) has countable Lebesgue spectrum. These flows display a strong form of *shearing* of nearby trajectories and were proved to be mixing by Kochergin in the 70’s, see [Ko75].

The main result of this paper concerns the nature of the spectrum of locally Hamiltonian flows on genus two surfaces, and, to the best of our knowledge, is the first general spectral result for surfaces of higher genus ($g \ge 2)$.

## Main Results

We now state the main result on the spectrum of locally Hamiltonian flows on genus two surfaces (see Sect. 1.1), as well as a result in the language of special flows from which it is deduced, see Sect. 1.2. The singularity criterion which is used to prove the first two results is stated (and proved) later in the paper, in Sect. 3 (as Theorem 3.1). In Sect. 1.3 we state a result on translation surfaces being well approximated by a single cylinder which is used as a key technical tool in the proof, but is also of independent interest, since it concerns Diophantine approximation-type questions for cylinders on translation surfaces in any genus (more precisely, any connected component of any stratum, see Sect. 1.3).

### Singularity of the spectrum of locally Hamiltonian flows in genus two

**Fig. 1:**
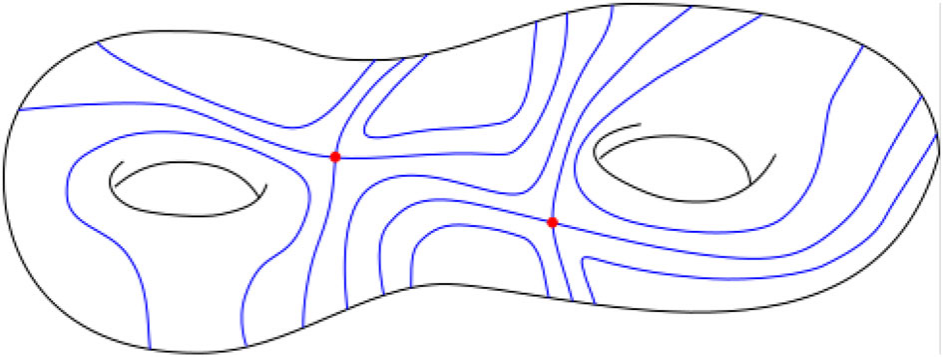
Trajectories of a locally Hamiltonian flow with two simple saddles on a surface of genus two

Throughout the paper let *M* denote a smooth, compact, connected, orientable surface and let $(\varphi _t)$ be a smooth flow preserving a smooth invariant measure (i.e. a measure with smooth positive density with respect to the area form on *M*). Equivalently, $(\varphi _t)$ is a *locally Hamiltonian flow*, see (Sect. 2.1). We assume that $(\varphi _t)$ has *non-degenerate* fixed points and is *minimal*. When the surface has genus $g=2$, this implies that there are *two* fixed points, both of which are *simple saddles* (i.e. four-pronged saddles, with two incoming and two outgoing separatrices), see Fig. 1. We will assume furthermore that the two saddles are *isomorphic* (in a sense specified in Sect. 2.3, see Definition 2.1).

#### Theorem 1.1

(Singular spectrum in genus two). A *typical* locally Hamiltonian flow on a surface *M* of genus two with two isomorphic saddles has *purely singular* spectrum.

Basic spectral notions, and in particular the definition of *singular spectrum*, are recalled in Sect. 3.1. The notion of *typical* used here is in a *measure theoretic sense* and it refers to a full measure set with respect to a natural measure class on locally Hamiltonian flows with given singularity types (sometimes referred to as the Katok fundamental class). For the definition of the measure class and the notion of typical used in the statement of Theorem 1.1, see Sect. 2.4 (and also more in general Sect. 2.2).

Theorem 1.1 is the first result on singularity of the spectrum of typical minimal locally Hamiltonian flows with non-degenerate singularities on surfaces in higher genus (and, to the best of our knowledge, the first general spectral result for smooth flows on surfaces of genus $g\ge 2$). We believe that the result is not only true in genus two, but in any genus $g\ge 2$. The importance of considering the case of genus two (to deal with some of key difficulties arising when passing from genus one to higher genus, or, in other words, from Poincaré sections which are rotations to interval exchange transformations), as well as the importance of the assumption that saddles are isomorphic for the strategy and techniques of proof will be explained in Sect. 1.4 below.

Singularity of the spectrum is in stark contrast with the recent result in [FFK] on flows on tori with a degenerated singularity (or stopping point), which are shown to have absolutely continuous (and actually countable Lebesgue) spectrum. It might be conjectured, from their result, that also in higher genus, in presence of sufficiently strong *degenerate* singular points, the spectrum is also absolutely continuous (and even countable Lebesgue). We remark that stopping points or non-degenerate fixed points (including centers) are known to produce mixing [Ko75] (at rates which are expected to be polynomial, see e.g. [Fa01]), while typical minimal locally Hamiltonian flows with *non-degenerate* saddles are known *not* to be mixing by the work of Scheglov [Sc] for genus two and Ulcigrai [Ul11] for any genus. At the heart of our proof is a strengthening of results on absence of mixing (in particular of the works [FL03, FL05] and [Sc]). When the flow is not minimal, and has non-degenerate singularities it has *several* minimal components, and the nature of the spectrum (for the restriction of a typical flow to a minimal component) is unclear. These flows are indeed mixing, but with sub-polynomial rate (see [Rav], which provides logarithmic upper bounds) and it is not clear whether to expect singularity or absolute continuity of the spectrum.

### Special flows with symmetric logarithmic singularites over symmetric IETs

Formally, Theorem 1.1 is deduced from a result for special flows (see below, or Sect. 2.5 for formal definitions). It is well known that any minimal (or minimal component of) locally Hamiltonian flow can be represented as *the special flow* over an *interval exchange transformations* or, for short, IET (see Sect. 2 for definitions and for the reduction). Our main result, that certain special flows have singular spectrum holds for IETs on *any* number of intervals in a special class (corresponding to *symmetric* permutations, or hyperelliptic strata). Let us give some definitions to formulate the precise statement.

An interval exchange transformation (IET) of *d* intervals $$T:I\rightarrow I$$ ($$I=[0,|I|)$$)^4^*with permutation* $$\pi $$ (on $$\{0,\dots , d-1\}$$) and *endpoints* (of the continuity intervals) $$0=:\beta _0< \beta _1< \dots \beta _{d-1}< \beta _d:= |I|$$ is a piecewise isometry which sends the interval $$I_i:=[\beta _i,\beta _{i+1})$$, for $$0\le i<d$$, by a translation, explicitly given by

$$\begin{aligned} T(x) = x-\beta _i +\beta _{\pi (i)}, \qquad \text {if }\ x \in [\beta _i,\beta _{i+1}). \end{aligned}$$

We say that $\pi :\{0,1,\dots , d-1\} \rightarrow \{0,1,\dots , d-1\}$ is *symmetric* if $\pi (i) = d-1-i$ for $0\le i <d$. Thus, in an IET with a symmetric permutation the order of the exchanged intervals is *reversed*. These are IETs which arise when considering (suitably chosen Poincaré sections of translation flows) *hyperelliptic strata* of translation surfaces, in particular for any genus $g \ge 1$ (see for example Lemma 2.1).

We say that a result holds *for almost every IET with permutation* $\pi$ if it holds for almost every choice of the lengths $|I_i|=\beta _{i+1}-\beta _i$ of the exchanged intervals (with respect to the restriction of the Lebesgue measure on $\mathbb {R}^d$ to the simplex $\Delta _{d-1} = \{(\lambda _1,\dots \lambda _d), \lambda _i\ge 0, \sum _{i=0}^{d-1} \lambda _i=1\}$).

**Fig. 2:**
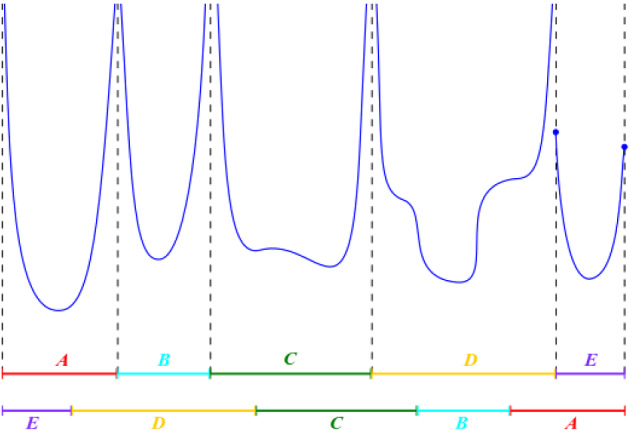
A special flow over a symmetric 5-IET with endpoints $\beta _0, \dots , \beta _5$ under a roof $f \in \mathcal {S}\text {ym}\mathcal {L}\text {og} \left( \sqcup _{i=0}^4 I_i \right)$. In this example $C_4=0$

The *special flow* over $T: I\rightarrow I$ under a positive, integrable roof function *f* (see also Sect. 2.5) is the vertical, unit speed flow on the region $X_f$ below the graph of *f*, given by $X_f := \{ (x,y) \in I\times \mathbb {R} : 0\le y < f(x) \}$, with the identification of each point on the graph, of the form (*x*, *f*(*x*)), where $x \in I$, with the base point (*T*(*x*), 0), as shown in Fig. 2 (see Sect. 2.5 for formal definitions).

We consider special flows under a *roof function* chosen in a class of (positive) functions which have *logarithmic singularities* at the discontinuities $\beta _i$. This is the type of singularities that arise in the special flow representation of locally Hamiltonian flows with simple saddles, see Sect. 2.7. More precisely, the class of functions, denoted by $\mathcal {S}\text {ym}\mathcal {L}\text {og} \left( \sqcup _{i=0}^{d-1}I_i \right)$ (to refer to *Symmetric Logarithmic* singularities), consists of positive real valued functions, defined on $\bigcup _{i=0}^{d-1} (\beta _i,\beta _{i+1})$ and such that the restriction $f\vert (\beta _i,\beta _{i+1})$ of *f* to each $(\beta _i,\beta _{i+1})$ is of the form

$$\begin{aligned} f\vert (\beta _i,\beta _{i+1}) = \left| C_i \log (x-\beta _i)\right| + \left| C_i \log (\beta _{i+1}-x)\right| + g_i(x) \end{aligned}$$

where $C_i \ge 0$ is a non-negative constant, $g_i$ is a function of bounded variation on $[\beta _i,\beta _{i+1}]$ and not all $C_i$ are simultaneously zero (see also the definitions in Sect. 2.6). Thus, if $C_i\ne 0$, *f* explodes logarithmically at each endpoint of $I_i$ and the *singularities* are *symmetric* (not necessarily $f\vert (\beta _i,\beta _{i+1})$ because of the presence of $g_i$). An example of a roof function is shown in Fig. 2.

The main result in the setting of special flows is the following.

#### Theorem 1.2

Let $\pi$ be a symmetric permutation. For almost every IET *T* with permutation $\pi$ and endpoints $\beta _i, 0\le i \le d$, for any $f\in \mathcal {S}\text {ym}\mathcal {L}\text {og} \left( \sqcup _{i=0}^{d-1} I_i \right)$, the special flow $(T_t^f)$ over *T* under *f* has purely singular spectrum.

The result in the context of special flows is hence more general (since it holds for IETs of any number $d\ge 2$ of exchanged intervals in the base), but unfortunately (similarly to the case of Scheglov’s result [Sc] on absence of mixing) this does not yield any general result for smooth locally Hamiltonian flows on surfaces of genus higher than two (see Remark 2.2). The role played by the symmetry of the IET, together with the symmetry in the roof, is explained in Sect. 1.4. We remark that the special case of Theorem 1.2 for $d=2$ recovers the main result from [FL03].

### Translation surfaces well approximated by single cylinders

We now state some results on cylinders in translation surfaces, which will be used as an ingredient in our proof of singularity of the spectrum but hold in more generality for any translation surface. As a reference on background material on translation surfaces, we refer the reader to one of the surveys [FoMa, Vi, Yo].

Let $(M, \omega )$ denote a (compact) translation surface, namely a Riemann surface *M* with an Abelian differential $\omega$ which defines a flat metric with conical singularities on *M*, which correspond to zeros of $\omega$. Recall that the notion of direction is well defined globally on a translation surface, thus directions can be identified with $S^1$. Denote by $\mathcal {C}yl_\omega$ the set of all cylinders in the translation surface $(M,\omega )$, i.e. $C\in \mathcal {C}yl_\omega$ is a maximal open annulus filled by homotopic simple closed (flat) geodesics. Any cylinder *C* is isometric to an annulus $J\times \mathbb {R}/c\mathbb {Z}$, where $J\subset \mathbb {R}$ is an (open) interval and $c>0$. The *core curve* of *C* is the closed geodesic represented by $\{x\}\times \mathbb {R}/c\mathbb {Z}$, where *x* is the mid point of the interval *J*.

For any cylinder $C\in \mathcal {C}yl_\omega$, denote by:

- $\gamma (C)$ the core curve of *C*;
- $\theta _C\in S^1$ the *direction* of *C* (i.e. the direction of the core curve $\gamma (C)$);
- *a*(*C*) the area of *C* with respect to the flat area-form induced by $\omega$;
- $\ell (C)>0$ the length of $\gamma (C)$ in the flat metric.

Assume that $a(M)=1$. For every $0<\epsilon <1$ let ${\mathcal {C}yl}^\epsilon _\omega$ be the subset of cylinders $C\in {\mathcal {C}yl}_\omega$ with $a(C)\ge 1-\epsilon$. We are interested in showing that on a typical translation surface, a full measure set of directions can be approximated (with a certain speed) by the directions of a sequence of cylinders ${\mathcal {C}yl}^\epsilon _\omega$, i.e. by single cylinders of area close to one.

To state the result, let $\mathcal {C}$ denote a connected component of (a stratum of) the moduli space of compact area one translation surfaces. In particular, all translation surfaces in $\mathcal {C}$ have the same number and type of conical singularities, or equivalently zeros of the Abelian differential. Let $m_\mathcal {C}$ denote the sum of the multiplicities of singular points (for example $m_\mathcal {C}=2$ for translation surfaces with genus two and two simple saddles, more in general $m_\mathcal {C}=\sum _{i=1}^n{\kappa _i}$ for connected components of the stratum $\mathcal {H}(\kappa _1, \dots , \kappa _n)$).

Recall that each $\mathcal {C}$ is endowed by a natural volume probability measure $\nu _\mathcal {C}$ (the Masur-Veech measure [Mas82, Ve]). Let $c_1(\mathcal {C})$ be the corresponding Siegel-Veech constant (we refer e.g. to [EsMa] for the notion of Siegel-Veech constant, which enters in counting problems on translations surfaces).

Let $\lambda$ denote the Lebesgue (probability) measure on the unit circle $S^1$ in the complex plane, which we freely identify with $[0,2\pi )$. The main result of this section is the following.

#### Proposition 1.3

(Directions well approximated by large cylinders). For $\nu _\mathcal {C}$-almost every translation surface $(M,\omega )\in \mathcal {C}$ and any $\epsilon >0$ there exists a sequence of cylinders $(C_i)_{i\ge 1}$ on $(M,\omega )$ so that $\ell ({C_i})\rightarrow +\infty$ as $i\rightarrow +\infty$ and for every $i\ge 1$ we have

$$\begin{aligned}a(C_i)\ge 1-\epsilon \quad \text {and}\quad \Vert \theta _{C_i}-\tfrac{\pi }{2}\Vert <\frac{1}{\ell ({C_i})^2 \log (\ell ({C_i}))}.\end{aligned}$$

The sequence of cylinders $(C_i)_{i\ge 1}$ gives what we will call a *good approximation* of the vertical direction by directions of single cylinders (when $\epsilon$ is small). The approximation rate $\ell ({C_i})^{-2} \log (\ell ({C_i}))^{-1}$ is chosen to allow us to prove singularity of the spectrum in the genus two case.

Proposition 1.3 can be easily deduced (see Sect. 5) from the following result on translation surfaces, which mimics, in the context of translation surfaces, the statement of Khintchine Theorem in Diophantine approximation.

#### Theorem 1.4

(Khintchine Theorem for cylinders on translation surfaces; c.f. [Ch, Theorem 1] and [MaTrWe, Theorem 6.1 (2)]). Let $\psi :\mathbb {R}^+ \rightarrow \mathbb {R}^+$ be non-increasing so that $t\psi (t)\le 1$ for *t* large enough and $\int _1^{+\infty } t \psi (t)=\infty$. Then for a.e. $(M,\omega )\in \mathcal {C}$ and every $0<\epsilon <1/2$ the set

1 $$\begin{aligned} W^\psi _\omega =\bigcap _{m\ge 1}\bigcup _{\{C \in {\mathcal {C}yl}^\epsilon _\omega :\ \ \ell (C)\ge m\}} \big \{\phi \in S^1: \Vert \theta _C-\phi \Vert <\psi (\ell ({C}))\big \} \end{aligned}$$

has full Lebesgue measure. Moreover, for a.e. $(M,\omega )\in \mathcal {C}$ there exists a sequence $(C_i)_{i\ge 1}$ in ${\mathcal {C}yl}_\omega ^\epsilon$ such that $\ell ({C_i})\rightarrow +\infty$ as $i\rightarrow +\infty$ and $\Vert \theta _{C_i}-\frac{\pi }{2}\Vert <\psi (\ell ({C_i}))$ for all $i\ge 1$.

This result’s proof is independent of the rest of the paper and follows from the methods of [Ch] and [MaTrWe]. It is proved in Sect. 5.

### Strategy of the proof of the main result

Let us conclude the introduction explaining the main ideas in the proof. To study ergodic and spectral properties of locally Hamiltonian flows, it is standard to exploit their representation as special flows over an IET (or a rotation when $g=1$). The growth of Birkhoff sums $S_n(f)= \sum _{k=0}^{n-1} f\circ T^k$ of the roof function *f* and its derivatives play a crucial role in the proof of properties such as mixing, weak mixing, multiple mixing, shearing properties and disjointness phenomena among others. Spectral behavior is no exception, but requires a *much* more delicate understanding of *weak limits* of Birkhoff sums.

The *criterion* we use for proving *singularity of the spectrum* of special flows (stated in Sect. 3.2) is devised to deal with flows which display *absence of mixing*. An important early criterion for absence of mixing appears in Katok’s work [Ka80], which shows that special flows over IETs under roof functions of bounded variation are never mixing, and by Kochergin’s, which shows the absence of mixing for special flows over rotations under a roof with a symmetric logarithmic singularity (see [Ko72, Ko07]). Both criteria require as input *tightness* of Birkhoff sums along some subsequences of *rigidity* (or *partial rigidity*) *times*, i.e. one has to show that there exists a sequence $$(q_n)$$ of times such that $$T^{q_n}$$ converges to identity on subsets $$E_n $$ of measure tending to one (if there is rigidity, or measure bounded below in the case of partial rigidity) and at the same time, for some centralizing sequence $$(a_n)$$ and uniform constant *C*, $$Leb\{ x\in E_n | \ |S_{q_n}(f)(x) - a_n|<C\}/Leb(E_n) \rightarrow 1 $$. In the case of rotations and functions of bounded variation, this follows easily from Denjoy-Kosma inequality, while for functions with symmetric logarithmic singularities one has to exploit a *cancellation* phenomenon among contributions coming from the symmetric singularities.

These type of criteria were pushed in two different directions in [FL03] and [Sc, Ul11]. Frączek and Lemańczyk in [FL03], considering the same example as Kochergin (special flows with one symmetric logarithmic singularity over rotations), showed that if, in addition to *tightness*, one can also control the *tails* of the distribution of the centralized Birkhoff sums $S_{q_n}(f)(x) - a_n$, one can prove much stronger results (using joinings and Markov operators) and deduce in particular spectral disjointness from mixing flows, which implies that the spectrum is purely singular. In [Sc, Ul11] IETs were considered on the base (which is required when treating surfaces of genus $g\ge 2$). In this case, cancellations are much more difficult to prove because of the absence of the Denjoy-Koksma inequality. To prove absence of mixing, though, it is sufficient to prove cancellations on carefully constructed partial rigidity times. The usual tool to study IETs (which is *not* used in this paper) is Rauzy-Veech induction, a renormalization algorithm for IETs. In [Ul11] Rauzy-Veech induction (and the log integrability of the associated cocycle) are heavily used to obtain cancellations at carefully chosen renormalization times. On the other hand, in Scheglov’s work [Sc], the cancellations were proved through a careful combinatorial analysis of the substitutions arising from the action of Rauzy-Veech induction on symmetric permutations. Ideally one would like to combine these two approaches in order to prove spectral results (as in [FL03]) for IETs (as in [Sc, Ul11]). The key difficulty is that cancellations are hard to achieve for IETs on sets of large measure (the cancellations in [Ul11] for example are crucially based on *balanced* Rauzy-Veech induction time, which are opposite to rigidity times).

In this paper, for surfaces of genus two or symmetric permutations, we (implicitly) exploit a very geometric approach to deduce cancellations, based on a simple mechanism which uses in an essential way the hyperelliptic involution: the key idea is that, for any symmetric (of equal backward and forward length) trajectory from a fixed point of the hyperelliptic involution, there are perfect cancellations for Birkhoff sums of the derivative of the roof function (see Sect. 4.2). Cancellations achieved through the hyperelliptic involution have the advantage of being compatible with rigidity. In particular, they can be shown to hold for Birkhoff sums along a rigidity tower of area close to one (i.e. a Rokhlin tower for the IET which comes from a cylinder of area close to one on the surface).

One of the advantages of this approach is that we do not make use at all of Rauzy-Veech induction. Theorem 1.1 also provides an independent proof of Scheglov’s work [Sc], which highlights the role played by the hyperelliptic symmetry in Scheglov’s combinatorial calculations.

In order to prove singularity of the spectrum using this approach (and the criterion stated in Sect. 3, which is a generalization of the criterion in [FL04, Corollary 5.2] and [FL03, Proposition 11]) though, another ingredient is needed, namely *good rigidity* (see Definition 4.2). Cancellations achieved thanks to the hyperelliptic involution only hold for Birkhoff sums along a full rigidity tower. To prove the exponential tails estimates needed to apply the criterion on the whole tower, one has to control incomplete sums, that can in general fail to be tight. These potentially worse estimates (see Remark 4.1) are compensated for by assuming that points in the base of the rigidity tower have a quantitatively good form of recurrence (see Definition 4.2). The existence of good rigidity towers for almost every IET is deduced (in Sect. 4.4) from the abundance of translation surfaces well approximated by single cylinders (i.e. from Proposition 1.3).

### Structure of the paper

In Sect. 2 we first recall some background material on locally Hamiltonian flows and their reduction to special flows, with particular attention to the form of the representation in the special case of genus two and two isomorphic saddles (see Lemma 2.1 and Corollary 2.2). Our criterion for singularity for special flows (Theorem 3.1) is stated and proved in Sect. 3.2, after recalling basic spectral notions in Sect. 3.1. Elementary but precise estimates on (Birkhoff sums of) functions with symmetric logarithmic singularities are proved in Sect. 4.1; these, combined with the symmetry and the cancellation arguments are explained in Sect. 4.2 (which follow from the hyperelliptic involution, see Lemmas 4.4 and 4.5), are then used in Sect. 4.4, in combination with the rigidity deduced from single cylinders (given by Proposition 1.3) to conclude the proof of the singularity result in genus two (i.e. Theorem 1.1). Finally, in Sect. 5 (which can be read independently), we prove the Khintchine-type result for translation surfaces (Theorem 1.4) and show how it implies Proposition 1.3 about translation surfaces well approximated by single cylinders.

## Locally Hamiltonian Flows and Reduction to Special Flows

In this section we recall some definitions, basic notions and background material on locally Hamiltonian flows (Sect. 2.1 and Sect. 2.2) and on special flows Sect. 2.5. We also quickly summarize some results in the literature of locally Hamiltonian flows Sect. 2.8.

### Smooth area-preserving flows as locally Hamiltonian flows

In this section we define locally Hamiltonian flows and show that they are equivalent to smooth area-preserving flows.

Assume that *M* is a 2-dimensional closed connected orientable smooth surface of genus $g\ge 1$. Let $X:M\rightarrow TM$ be a smooth tangent vector field with finitely many fixed points and such that the corresponding flow $(\varphi _t)_{t\in \mathbb {R}}$ preserves a smooth volume form $\omega$ (which is locally given by $V(x,y) d x \wedge d y$ for some smooth positive real valued function $V:U \rightarrow \mathbb {R}$ on the coordinate chart). Then, letting $\eta :=\imath _X\omega =\omega ( \eta , \,\cdot \,)$, where $\imath _X$ denotes the contraction operator, we have $d\eta =0$. Furthermore, since $\eta$ is a smooth closed 1-form, for any $p\in M$ and any simply connected neighbourhood *U* of *p* there exists a smooth (local Hamiltonian) map (unique up to additive constant) such that $dH=\eta$ on *U*.

Conversely, let $(M, \omega )$ be a 2-dimensional symplectic manifold, where *M* is a closed connected orientable smooth surface of genus $g\ge 1$ endowed with the standard area form $\omega$ (obtained as pull-back of the area form ${d} x \wedge {d} y$ on $\mathbb {R}^2$). Let $\eta$ be a smooth closed real-valued differential 1-form. Let *X* be the vector field determined by $\eta = \imath _X \omega$ and consider the flow $(\varphi _t)_{t\in \mathbb {R}}$ on *M* associated to *X*. Since $\eta$ is closed, the transformations $\varphi _t$, $t \in \mathbb {R}$, are area-preserving (i.e. preserve the area form $\omega$ and the measure given by integrating it). We will always assume that the form is normalized so that the associated measure gives area 1 to *M*.

The flow $(\varphi _t)_{t\in \mathbb {R}}$ is known as the *multi-valued Hamiltonian* flow associated to $\eta$. Indeed, the flow $(\varphi _t)_{t\in \mathbb {R}}$ is *locally Hamiltonian*, i.e. *locally* one can find coordinates (*x*, *y*) on *M* in which $(\varphi _t)_{t\in \mathbb {R}}$ is given by the solution to the equations ${\dot{x}}={\partial H}/{\partial y}$, ${\dot{y}}=-{\partial H}/{\partial x}$ for some smooth real-valued Hamiltonian function *H*. A *global* Hamiltonian *H* cannot be in general defined (see [NiZh], §1.3.4), but one can think of $(\varphi _t)_{t\in \mathbb {R}}$ as globally given by a *multi-valued* Hamiltonian function.

**Fig. 3:**
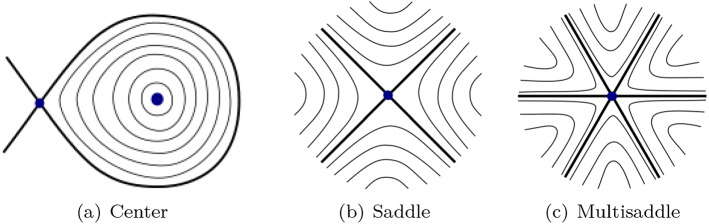
Type of non-degenerate fixed points for an area-preserving flow

When $g\ge 2$, the (finite) set of fixed points of $(\varphi _t)_{t\in \mathbb {R}}$ is always non-empty. We will always assume that the 1-form $\eta$ is *Morse*, i.e. it is locally the differential of a Morse function. Thus, zeros of $\eta$ are isolated and finite and all correspond to either centers (see Fig. 3a) or simple saddles (see Fig. 3b), see Sect. 2.3 (as opposed to degenerate *multi-saddles* which have 2*k* separatrices for $k>2$, see Fig. 3c).

### Topology and measure class on locally Hamiltonian flows

One can define a *topology* on locally Hamiltonian flows by considering perturbations of closed smooth 1-forms by smooth closed 1-forms. With respect to this topology, the set of locally Hamiltonian flows whose zeros are all Morse (hence isolated and finite, simple saddles or centers) is open and dense (and hence in particular *generic* in the Baire category sense), see for example Lemma 2.3 in [Rav]. Let $\Sigma$ be the set of fixed points of $\eta$ and let *k* be the cardinality of $\Sigma$.

The *measure-theoretical notion of typical* that we use is defined as follows and coincide with the notion of typical induced by the *Katok fundamental class* (introduced by Katok in [Ka73], see also [NiZh]). We recall that two measures belong to the same measure class if they have the same sets of zero mesure (and hence induce the same notion of full measure, or *typical*)); thus, a *measure class* is uniquely identified by a collection of sets which have measure zero with respect to all measures in the class.

Let $\gamma _1, \dots , \gamma _n$ be a base of the relative homology $H_1(M, \Sigma , \mathbb {R})$, where $n=2g+k-1$ ($k:=\#\Sigma$). The image of $\eta$ by the period map *Per* is $Per(\eta ) = (\int _{\gamma _1} \eta , \dots , \int _{\gamma _n} \eta ) \in \mathbb {R}^{n}$. The pull-back $Per_* Leb$ of the Lebesgue measure class by the period map gives a measure class on closed 1-forms (with *k* critical points): explicitely, the measure zero sets for this measure class are all preimages through *Per* of measure zero sets in $\mathbb {R}^{n}$ (with respect to the Lebesgue measure *Leb* on $\mathbb {R}^{n}$). We say that a property is *typical* if it is satisfied for a set of locally Hamiltonian flows a *full measure*, namely the complement of a measure zero set for this measure class.

A *saddle connection* is a flow trajectory from a saddle to a saddle and a *saddle loop* is a saddle connection from a saddle to the same saddle (see Fig. 3a). Notice that if the set of fixed points $\Sigma$ contains a center, the island of closed orbits around it is automatically surrounded by a saddle loop homologous to zero (see Fig. 3a). The set of locally Hamiltonian flows which have at least one saddle loop *homologous to zero* form an *open* set.^5^ Flows in this open set decompose into several *minimal components*.^6^ On the other hand, in the open set $\mathscr {U}_{min}$ consisting of locally Hamiltonian flows with only simple saddles and no saddle loops homologous to zero a typical flow (in the measure theoretical sense defined above) has no saddle connections and hence it is *minimal* by a result of Maier [Mai] (or, in the language of special flows introduced in the next section, by the result of Keane [Kea] on IETs).

#### Remark 2.1

Minimal locally Hamiltonian flows (as well as minimal components) can be seen as (singular) time-reparametrizations of *translation flows* (linear flows on translation surfaces), i.e. they have the same orbits as a translation flow, but the movement along the orbits happens with different speed (and in particular it takes an infinite time to reach saddles). This follows for example from a result in [Mai], which guarantees that any 1-form $\eta$ without saddle loops homologous to zero is the real part of a holomorphic one form (see [Zor]).

### Singularities and normal forms

In this section we associate an invariant to each non-degenerate fixed point which will play a crucial role in describing isomorphic singularities and their special flow representation.

Let *M* be an *m*-dimensional $C^2$-manifold equipped with a volume form $\omega$. Let $f:M\rightarrow \mathbb {R}$ be a $C^2$-map whose critical points are isolated. Suppose that $p\in M$ is a critical point of *f* and let us consider local coordinates $(x_1,\ldots , x_m)$ in a neighbourhood of *p* so that $(0,\ldots ,0)$ are local coordinates of *p*. In these local coordinates $\omega _{(x_1,\ldots , x_m)}=V(x_1,\ldots , x_m)\,dx_1\wedge \ldots \wedge d x_m$, where *V* is a positive (or negative) function. Let

$$\begin{aligned}K_\omega (f,p):=\frac{\det {\text {Hess}}(f)(0,\ldots ,0)}{V^2(0,\ldots ,0)}.\end{aligned}$$

Since $df(p)=0$, $K_\omega (f,p)$ does not depend on the choice of local coordinates and it imitates the notion of curvature (on the graph of *f*) at any critical point of *f* even if *M* is not equipped with any Riemannian metric. Moreover, $K_\omega (f,p)\ne 0$ if and only if *p* is a non-degenerate critical point and by the Morse lemma there exist local coordinates $(x_1,\ldots , x_m)$ in a neighbourhood of *p* such that

$$\begin{aligned}f(x_1,\ldots , x_m)=f(0,\ldots ,0)-x^2_1-\ldots -x^2_k+x^2_{k+1}+\ldots +x^2_m\end{aligned}$$

and $${\text {sgn}}K_\omega (f,p)=(-1)^k$$.

Assume now that *M* is two dimensional and consider a local Hamiltonian $H: U \rightarrow \mathbb {R}$, $U \subset M$, of a locally Hamiltonian flow $(\varphi _t)_{t\in \mathbb {R}}$ preserving the area form $\omega$. If *p* is a fixed point of $(\varphi _t)_{t\in \mathbb {R}}$ (hence a critical point of *H*), then we can define

$$\begin{aligned}K_{\omega ,X}(p):=K_\omega (H,p).\end{aligned}$$

The quantity $K_{\omega ,X}(p)$ does not depend on the choice of local Hamiltonian, hence it is well defined.

A fixed point *p* is *non-degenerate* exactly when $K_{\omega ,X}(p)\ne 0$. If $K_{\omega ,X}(p)>0$ then *p* is the centre of a topological disc filled with periodic orbits, as in Fig. 3a. If $K_{\omega ,X}(p)<0$ then *p* is a saddle point (see Fig. 3b).

### Isomorphic saddles

We will use the following working definition of *isomorphic* (simple) *saddles*.

#### Definition 2.1

*(Isomorphic saddles).* We say that two saddles corresponding to fixed points $p_1,p_2$ of $(\varphi _t)_{t\in \mathbb {R}}$ are *isomorphic* iff $K_\omega (H,p_1)= K_\omega (H,p_2)$.

Indeed, the above definition implies that, for both $i=1,2$, we can find local coordinates around $p_i$ so that $p_i$ is mapped to (0, 0), $\omega$ is given by the standard form $dx \wedge dy$, and the local Hamiltonian has the form $H (x,y) = K x y + \text {higher order terms},$ for a common value $K:= \sqrt{-K_\omega (H,p_1)}=\sqrt{-K_\omega (H,p_2)}$. This property is satisfied if there is a smooth symplectic (preserving $\omega$) isomorphism mapping flow trajectories to flow trajectories among the two local neighbours.

#### Definition 2.2

*(Isomorphic saddles locus).* We will denote by $\mathcal {K}$ the set of locally Hamiltonian flows on a surface of genus two in $\mathcal {U}_{min}$ which have two isomorphic simple saddles.

The notion of *typical* on $\mathcal {K}\subset \mathcal {U}_{min}$ (which is the notion used in the statement of Theorem 1.1) is obtained restricting the notion of Katok measure class (see Sect. 2.2) to $\mathcal {K}$ as follows. Consider the period map $Per: \mathcal {K}\rightarrow \mathbb {R}^n$ obtained restricting the period map $Per: \mathcal {U}_{min}\rightarrow \mathbb {R}^n$ defined in Sect. 2.2 to $\mathcal {K}\subset \mathcal {U}_{min}$. We say that a property holds for a *typical* flow in $\mathcal {K}$ if it *fails* on a set of measure zero with respect to the pull back of the Lebesgue measure class via $Per: \mathcal {K}\rightarrow \mathbb {R}^n$, namely it fails on the preimage $Per^{-1}(Z)$ of a set $Z\subset \mathbb {R}^n$ with $Leb(Z)=0$. See also Remark 2.3 for a reformulation of this notion of typical in terms of special flows representations.

### Special flows

Let us now recall the definition of *special flow*. Let *T* be an automorphism of a standard (Borel) probability space $(X,\mathcal {B},\mu )$. Let $f:X\rightarrow \mathbb {R}_{>0}$ be an integrable function so that $\inf _{x\in X} f(x)>0$. Let us denote by $S_n(f)(x)$ the *Birkhoff sum* defined by

$$\begin{aligned}S_n(f)(x)=\left\{ \begin{array}{lll} \sum _{0\le i<n}f(T^ix)&{}\quad \text {if}&{} n\ge 0\\ -\sum _{n\le i<0}f(T^ix)&{}\quad \text {if}&{} n< 0. \end{array} \right. \end{aligned}$$

The *special flow* $(T^f_t)_{t\in \mathbb {R}}$ built *over* the automorphism *T* and *under* the *roof function* *f* acts on

$$\begin{aligned}X^f:=\{(x,r)\in X\times \mathbb {R}: 0\le r<f(x)\}\end{aligned}$$

so that

$$\begin{aligned}T^f_t(x,r)=(T^nx,r+t-S_n(f)(x)),\end{aligned}$$

where $n=n(t,x) \in \mathbb {Z}$ is a unique integer number with $S_n(f)(x)\le r+t<S_{n+1}(f)(x)$. Under the action of $(T^f_t)_{t>0}$, a point $(x,y) \in X_f$ moves with unit velocity along the vertical line up to the point (*x*, *f*(*x*)), then jumps instantly to the point $\left( T(x),0 \right)$, according to the base transformation and afterward it continues its motion along the vertical line until the next jump and so on. The integer *n*(*t*, *x*) (for $t>0$) is the number of discrete iterations of the map *T* undergone by the orbit of *x* up to time *t*.

The flow $(T^f_t)_{t\in \mathbb {R}}$ preserves the finite measure $\mu ^f$ which is the restriction of $\mu \times \lambda _\mathbb {R}$ to $X^f$. If *T* is ergodic with respect to $\mu$, it is easy to see then $(T^f_t)_{t\in \mathbb {R}}$ is also ergodic (with respect to $\mu ^f$), see e.g. [CFS].

### Roofs with logarithmic singularities

We now define the class of functions which we work with and arise as roof functions of locally Hamiltonian flows with non-degenerate saddles.

Let *T* be an IET with endpoints of the continuity intervals $0:=\beta _0<\beta _1< \dots \beta _{d-1}<\beta _d:=|I|$ (see Sect. 1.2).

#### Definition 2.3

(logarithmic singularities). We say that a function *f* has *pure logarithmic singularities* at the endpoints $\beta _i$ of *T* and write $f \in \mathcal {L}\text {og}^\text {p} \left( \sqcup _{i=0}^{d-1} I_i \right)$ if it is of the form

2 $$\begin{aligned} f(x)=\sum _{0\le i<d}\chi _{(\beta _i,\beta _{i+1})}(x) \big (-C_i^+\log (x-\beta _i)-C_{i+1}^-\log (\beta _{i+1}-x)\big ), \end{aligned}$$

for some constants $C_i^{\pm }\ge 0$, not all simultaneously zero. Notice that the signs are chosen so that $f\ge 0$.

We say that *f* has pure *symmetric* logarithmic singularities at the endpoints $\beta _i$ of *T* and write $f \in {\mathcal {S}\text {ym}\mathcal {L}\text {og}^\text {p} \left( \sqcup _{i=0}^{d-1} I_i \right) }$ if in addition we have that $C_i^+=C_{i+1}^-$ for $0\le i< d$, so that the function is *symmetric* on each interval $(\beta _i,\beta _{i+1})$.

We say that *f* has *logarithmic singularities* (resp. *symmetric logarithmic singularities*) and write $f \in \mathcal {L}\text {og} \left( \sqcup _{i=0}^{d-1} I_i \right)$ (resp. $f \in \mathcal {S}\text {ym}\mathcal {L}\text {og} \left( \sqcup _{i=0}^{d-1} I_i \right)$) if and only if *f* can be written as $f=f^p+g$ where $f^p \in \mathcal {L}\text {og}^\text {p} \left( \sqcup _{i=0}^{d-1} I_i \right)$ (resp. $f \in \mathcal {S}\text {ym}\mathcal {L}\text {og}^\text {p} \left( \sqcup _{i=0}^{d-1} I_i \right)$) has pure logarithmic (symmetric) singularities and $g:I\rightarrow \mathbb {R}$ is a function of bounded variation.

We remark that we allow some of the $C_i^\pm$ to be zero; so *f* could have a finite one-sided limit at some $\beta _i$ (but we assume that at least one of the singularities is indeed logarithmic). We notice also that this symmetry condition (which is symmetric on *each* exchanged interval) is not the same than appears in other works on locally Hamiltonian flows with non-degenerate saddles (where symmetric logarithmic singularities refers to functions in ${\mathcal {L}\text {og} \left( \sqcup _{i=0}^{d-1} I_i \right) }$ such that $\sum _{i=0}^{d-1}C_i^+= \sum _{i=1}^{d}C_i^-$). We will use the assumption that the saddles are isomorphic in Theorem 1.1 to obtain this stronger form of symmetry for such (genus 2) surfaces.

### Reduction to symmetric special flows

It is well known that minimal (or minimal components of) locally Hamiltonian flows can be represented as special flows over rotations (in genus one) or interval exchange transformations (for $g\ge 2$). The roof function has a finite number of singularities (where it explodes to infinity) which are of *logarithmic-type* (see the form of singularities in Definition 2.3) if the fixed points are simple saddles or *power-type* singularities (i.e. singularities of the form $C_i^{\pm }/\vert x-\beta _i \vert ^{\alpha _i}$ for some power $0<\alpha _i<1$) in presence of (degenerate) multi-saddles (as in Fig. 3c) or stopping points. In case of minimal flows with only simple saddles (or more in general when there are no saddle loops homologous to zero), the logarithmic singularities display a form of *symmetry*.^7^ For Theorems 1.1 and 1.2 we require a stronger form of symmetry for both the roof and the base transformation.

The following Lemma provides the reduction to symmetric special flows which we need to prove the result on flows in genus two (see in particular Corollary 2.2). While (*ii*) is standard (and included only for completeness), (*i*) and (*iii*) provide the required more detailed information on the symmetry of the base and the roof (in particular the precise values of the constants $C_i^\pm$).

#### Lemma 2.1

(Symmetries of the reduction to special flows). Let $(\varphi _t)_{t \in \mathbb {R}}$ be a *minimal* locally Hamiltonian flow on a surface *M*. Then, $(\varphi _t)_{t \in \mathbb {R}}$ is *measurably isomorphic* to a special flow $(T^f)_{t \in \mathbb {R}}$ over an IET *T* (whose endpoints are denoted by $\beta _i$, $0 \le i \le d$) under a roof function $f:I \rightarrow \mathbb {R}_{>0}\cup \{ + \infty \}$. The special flow representation can be chosen to that:

(i) if *M* has genus 1 or 2 then *T* is a *d*-IET given by a *symmetric permutation* (with $d= 2g$ if there is a unique saddle or $d=2g+1$ if there are two);
(ii) when $(\varphi _t)_{t \in \mathbb {R}}$ has only *simple* saddles, $f\in \mathcal {L}\text {og} \left( \sqcup _{i=0}^{d-1}I_i \right)$;
(iii) under the assumptions of (*ii*), the constants $C_i^\pm$ in (2) are given by the values of the invariants $K_{\omega ,X} (p)$ associated to saddle points: if the forward $(\varphi _t)_{t \in \mathbb {R}}$-orbit of $\beta _i$ meets the saddle point *p* before returning to *I*, then
  3 $$\begin{aligned} C_i^+=C_{i}^-=\frac{1}{\sqrt{-K_{\omega ,X}(p)}}.\end{aligned}$$

The proof of Lemma 2.1 is presented below. Combining $(i)-(iii)$ of Lemma 2.1, we have the following Corollary which we will use to prove Theorem 1.1.

#### Corollary 2.2

If *M* has genus two and $(\varphi _t)_{t \in \mathbb {R}}$ is a minimal locally Hamiltonian flow with two isomorphic simple saddles $p_1,p_2\in M$, then it is isomorphic to a special flow over an IET *T* with a symmetric permutation $\pi$ with $d=5$ and roof $f\in \mathcal {S}\text {ym}\mathcal {L}\text {og} \left( \sqcup _{i=0}^{4}I_i \right)$. More precisely, there exists $0\le i_0<5$ such that $C^+_{i_0}=C^-_{i_0+1}=0$ and

$$\begin{aligned}C_i^+ =C_{i+1}^-=\frac{1}{\sqrt{-K_{\omega ,X}(p_1)}}=\frac{1}{\sqrt{-K_{\omega ,X}(p_2)}}>0\quad \text { for all }\quad i\ne i_0.\end{aligned}$$

#### Remark 2.2

The conclusion of Part (*i*) of Lemma 2.1 also holds more in general when the flow $(\varphi _t)_{t \in \mathbb {R}}$ has one or two saddles and is the time-change of a linear flow on a translation surface *M* in a hyperelliptic component of stratum of the form $\mathcal {H}(2g-2)$ or $\mathcal {H}(g-1,g-1)$, $g\ge 1$ (the saddles then have respectively $4g-2$ or 2*g* and 2*g* separatrices). On the other hand, to have a roof $f\in \mathcal {S}ym\mathcal {L}og$ one needs by Part (*ii*) to have only simple saddles (with 4 separatrices), so this forces $g=2$ and two singularities. Thus, the special flows in Theorem 1.2 arise as special representation of *minimal* smooth surface flows only in genus two. Finally, notice also that the assumption that the two saddles are isomorphic is needed to have the symmetry of the constants.

#### Proof of Lemma 2.1

Since $(\varphi _t)_{t\in \mathbb {R}}$ is minimal, any curve $\gamma$ transverse to $(\varphi _t)_{t\in \mathbb {R}}$ is a *global* transversal (i.e. intersects all infinite orbits) and hence provides a Poincaré section for $(\varphi _t)_{t\in \mathbb {R}}$. Let us say that the parametrization of $\gamma$ is *standard* if $\gamma :I\rightarrow M$ (where *I* is an interval starting at zero) is parametrized so that $\eta (d\gamma )=1$. It is well known (see for example [Yo, Section 4.4]) that, in the standard parametrization, the Poincaré first return map $T:I\rightarrow I$ to $\gamma$ is an IET. The number of exchanged intervals is $d=2g+k-1$ (where *k* is the cardinality of the set of fixed points) if the endpoints of $\gamma$ are chosen on separatrices and, if $0=\beta _0<\beta _1<\ldots <\beta _d=|I|$ denote the endpoints of exchanged intervals, the (forward) trajectories from all the $\beta _i$’s are separatrices which end in a saddle (notice that there are no centers since $(\varphi _t)_{t\in \mathbb {R}}$ is minimal) and do not return to *I*, with the exception of two of them, which first return to the endpoints of $\gamma$ or its backward trajectory is a separatrix which starts from a saddle.

To prove (*i*), it is convenient to recall that $(\varphi _t)_{t\in \mathbb {R}}$ is a time-change of a translation flow $(h_t)_{t\in \mathbb {R}}$ (see Remark 2.1). Let us denote by $(M, \omega )$ the translation structure on *M*. If *M* has genus one or two, then it belongs to one of the strata $\mathcal {H}(0),$ $\mathcal {H}(2)$ or $\mathcal {H}(1,1)$ and it admits a *hyperelliptic involution*, i.e. there is a diffeomorphism $\iota : M \rightarrow M$ (affine in the coordinate charts of $(M, \omega )$) such that $\iota ^2 =Id$. Let us choose $\gamma$ so that $\gamma (I)$ is an interval in $(M,\omega )$ and the image $\gamma (x_0)$ of the midpoint $x_0=\vert I \vert /2$ of *I* is a *Weierstrass point*, i.e. a fixed point of $\iota$, i.e. $\iota (\gamma (x_0))=\gamma (x_0)$. Thus $\iota$ fixes $\gamma$: let us denote $S: I \rightarrow I$ the symmetry such that $\iota (\gamma (x)) = \gamma (S(x))$. Moreover, $\iota$ inverts the direction of trajectories of $(h_t)_{t\in \mathbb {R}}$, so that we have $h_{t}(\iota (q)) =\iota (h_{-t}(q))$ for all $q \in \gamma , t>0$. Observe that this implies that the *backward* trajectory from *q* first returns to $\gamma$ in *p* iff the *forward* trajectory from $\iota (q)$ first return to $\gamma$ in $\iota (p)$.

Let $$q\in \gamma $$ be the first return of the forward trajectory of $$p\in \gamma $$ to $$\gamma $$; if $$x,y\in I$$ are such that $$\gamma (x)=p, \gamma (y)=q$$, since *T* is by definition the first return map in the coordinates on *I*, this means that $$T(x) = y$$. Remark that equivalently *p* is the first return of the *backward* trajectory from *q* to $$\gamma $$. Applying $$\iota $$, $$\iota (p),\iota (q)$$ have coordinates respectively *S*(*x*), *S*(*y*) and, by the observation in the previous paragraph, the first return of the forward trajectory from $$\iota (q)$$ to $$\gamma $$ is $$\iota (p)$$ (since *p* as just remarked is the first backward return of *q*). In coordinates, this can be written as $$ T (S(y)) = S(x)$$. Combining both equations in coordinates and recalling that $$S^2=id$$, we get

4 $$\begin{aligned} S(x) = T(S(y)) = T(S (T(x))) \qquad \Leftrightarrow \qquad T(x)= S\circ T^{-1}\circ S(x), \end{aligned}$$

for all $x \in I$. Since in the translation structure $S:I \rightarrow I$ is an affine symmetry and it fixes $x_0=\vert I \vert /2$, *S* must be of the form $S(x)= \vert I \vert -x$. One can then show that (4) forces the *T* to be symmetric, i.e. the permutation $\pi$ must reverse the order of the intervals. This concludes the proof of (*i*).

By standard ergodic theory (see e.g. [CFS]), $(\varphi _t)_{t\in \mathbb {R}}$ is metrically isomorphic to the special flow over its Poincaré section *T* under the function *f* given by the first return time. If all saddles are *simple*, by the local form of Hamiltonian saddles (as first remarked by Arnold in [Arn], see also [CF, § 7.1]) the first return time function $f:I\rightarrow \mathbb {R}_{>0}\cup \{+\infty \}$ is given by $f\in \mathcal {L}\text {og} \left( \sqcup _{i=0}^{d-1} I_i \right)$, i.e. it has the form

$$\begin{aligned}f(x)=\sum _{0\le i<d}\big (-C_i^+\log (x-\beta _i)-C_{i+1}^-\log (\beta _{i+1}-x)\big )\chi _{(\beta _i,\beta _{i+1})}(x)+g(x),\end{aligned}$$

where $g:I\rightarrow \mathbb {R}$ is of bounded variation. This concludes the proof of (*ii*).

Let us now show that if $\gamma (\beta _i)$ is the first backward hitting point of a separatrix incoming to a saddle *p* to $\gamma (I)$ then (*iii*) hold. Choose local coordinates (*x*, *y*) in a neighbourhood *U* of *p* and a local Hamiltonian so that $H(x,y)=xy$. Then $\omega (x,y)=V(x,y)dx\wedge dy$, where *V* is a positive (or negative) smooth map. Fix $\varepsilon >0$ such that $[-\varepsilon ,\varepsilon ]\times [-\varepsilon ,\varepsilon ]\subset U$. In local coordinates the differential equation associated with the vector field *X* is given by

$$\begin{aligned}x'=\frac{x}{V(x,y)},\; y'=-\frac{y}{V(x,y)}\qquad \text { and }\quad t \mapsto H(x(t),y(t))=x(t)y(t)\quad \text { is constant.}\end{aligned}$$

Therefore the forward semiorbit of any $\pm (x/\varepsilon ,\varepsilon )$ with $x\in [-\varepsilon ^2,\varepsilon ^2]\setminus \{0\}$ leaves the square $[-\varepsilon ,\varepsilon ]\times [-\varepsilon ,\varepsilon ]$ at $\pm {\text {sgn}}(x)(\varepsilon , x/\varepsilon )$. Moreover, the time it takes to go through the square is

$$\begin{aligned} \tau (x)&=\int _0^{\tau (x)}dt=\int _0^{\tau (x)}\frac{V(x(t),x/x(t))x'(t)}{x(t)}dt\\&=\int _{|x|/\varepsilon ^2}^{1}\frac{V\big (\pm \varepsilon \big ({\text {sgn}}(x) s,\frac{|x|/\varepsilon ^2}{s}\big )\big )}{s}ds. \end{aligned}$$

To get the last equality we use the substitution $x(t)=\pm {\text {sgn}}(x)\varepsilon s$. By Lemma A.1 in [FU], $\tau (x)=-V(0,0)\log x+g(x)$, where $g:[-\varepsilon ^2,\varepsilon ^2]\rightarrow \mathbb {R}$ is of bounded variation. Let us consider the transversal curves $\gamma :[-\varepsilon ^2,\varepsilon ^2]\rightarrow M$ given by $\gamma (s)=\pm (s/\varepsilon ,\varepsilon )$ or $\gamma (s)=\pm (\varepsilon ,s/\varepsilon )$. Since $\eta$ in local coordinates is given by $\eta _{(x,y)}=y\,dx+x\,dy$, we always have $\eta (d\gamma )=1$ so all of them are standard. As

$$\begin{aligned}K_{\omega ,X}(p)=\frac{\det {\text {Hess}}(H)(0,0)}{V^2(0,0)}=\frac{-1}{V^2(0,0)},\end{aligned}$$

we have $V(0,0)=1/{\sqrt{-K_{\omega ,X}(p)}}$. This completes the proof of (*iii*) and hence of the Lemma. $\square$

#### Proof of Corollary 2.2

By Lemma  2.1 one can choose the special representation of $(\varphi _t)_{t \in \mathbb {R}}$ so that (by (*i*), since $(\varphi _t)_{t \in \mathbb {R}}$ has two saddles) $\pi$ is symmetric with $d=2g+1=5$ and furthermore, since the saddles are both simple, by (*ii*), $f\in \mathcal {L}\text {og} \left( \sqcup _{i=0}^{4}I_i \right)$.

Suppose that the forward $(\varphi _t)_{t \in \mathbb {R}}$-orbit (or equivalently $(h_t)_{t\in \mathbb {R}}$-orbit) of $\beta _i$ ($0\le i<5$) meets the saddle point *p* before returning to *I*. Applying the involution $\iota$, we obtain that the backward $(h_t)_{t\in \mathbb {R}}$-orbit of $S\beta _i=|I|-\beta _i$ meets the saddle point $\iota (p)$ before backward returning to *I*. Since *T* transforms $(\beta _i,\beta _{i+1})$ on $(|I|-\beta _{i+1},|I|-\beta _i)$ by a translation, it follows that the forward $(h_t)_{t \in \mathbb {R}}$-orbit (or equivalently $(\varphi _t)_{t\in \mathbb {R}}$-orbit) of $\beta _{i+1}$ meets the saddle point $\iota (p)$ before returning to *I*. By (*iii*) in Lemma 2.1 and the fact that *p* and $\iota (p)$ are isomorphic, we have

$$\begin{aligned}C_i^+=\frac{1}{\sqrt{-K_{\omega ,X}(p)}}=\frac{1}{\sqrt{-K_{\omega ,X}(\iota (p))}}=C^-_{i+1}.\end{aligned}$$

The same argument shows that if the forward $(\varphi _t)_{t \in \mathbb {R}}$-orbit of $\beta _i$ does not meet any saddle point before returning to *I* then $\beta _{i+1}$ satisfies the same property. Then $C^+_{i}=C^-_{i+1}=0$. By the proof of (*i*) in Lemma 2.1, $\beta _i$ and $\beta _{i+1}$ are the only two points satisfying this property, which completes the proof. $\square$

#### Remark 2.3

In the reduction described above of a locally Hamiltonian flow to a special flow over an IET *T*, one can see that the length of each interval $(\beta _i, \beta _{i+1})$ exchanged by *T* coincide with one of the coordinates of $Per(\eta )$, where we recall that *Per* denotes the period map defined in Sect. 2.2. Thus, for every subset $\mathcal {U}\subset \mathcal {U}_{min}$ of locally Hamiltonian flows, the set $\{ Per (\eta ), \eta \in \mathcal {U}\}$ has full Lebesgue measure as long as a full measure set of IET on *d* intervals and fixed permutation (with respect to the Lebesgue measure on the lenghts of the intervals) appears in the base of special flows representations of flows in $\mathcal {U}$.

Furthermore, to show that a property is *typical* within a subset $\mathcal {U}\subset \mathcal {U}_{min}$ of locally Hamiltonian flows (in the sense of Sect. 2.4 for $\mathcal {U}=\mathcal {K}$), it is sufficient to show that it holds for *every* special flow representation of a flow in $\mathcal {U}$ over a full measure set of IETs in the base (in this way it can only fail only on the preimage via *Per* of a zero Lebesgue mesure set). In particular, to show that singularity of the spectrum holds for a typical flow in the isomorphic saddle locus $\mathcal {K}$ (recall Definition 2.2), it is enough to show that for almost every IET with a symmetric permutation $\pi$ with $d=5$, every special flow with symmetric logarithmic singularities $f\in \mathcal {S}\text {ym}\mathcal {L}\text {og} \left( \sqcup _{i=0}^{4}I_i \right)$ has singular spectrum.

### Previous results on ergodic and spectral properties

Let us briefly summarize the mixing and spectral results known for locally Hamiltonian flows and special flows over rotations and IETs. Mixing properties of locally Hamiltonian flows turn out to depend crucially on the type of singularities (i.e. fixed points) of the flow.

**Flows with no singularities or degenerate singularities.** If a smooth flow on a compact surface has no singularities, by Poincaré-Hopf theorem the surface has genus one and hence is a torus. It is well known that such smooth flows on the torus are not mixing. Furthermore, let us point out that Katok in [Ka80] showed that linear flows on translation surfaces (and more in general special flows over IETs -thus in particular over rotations- under a roof function of *bounded variation*) are *never* mixing.

On the torus, one can introduce a fake singularity by adding a stopping point. This operation can drastically change the ergodic and spectral properties: as already mentioned in the introduction, Forni, Fayad and Kanigowski recently showed in [FFK] that if the stopping point is sufficiently strong, the resulting flow has countable Lebesgue spectrum. Stopping points can be thought as *degenerate* saddles, with only two separatrices. On a surface of any genus $g\ge 1$, the presence of either a stopping point, or more in general, of a *degenerate* critical points (which correspond to *multi-saddles*, i.e. saddles with 2*n* prongs, $n\ne 2$ integer, see Fig. 3c) produce *power-like* singularities in the special flow representation. Special flows over IETs with these type of singularities are mixing (for a full measure set of base transformations) by Kochergin’s work [Ko75].

**Flows with logarithmic singularities over rotations.** Singularities which are *non-degenerate*, as we just saw (in the previous Sect. 2.7) give rise to special flows with *logarithmic* singularities. In this case, mixing depends on the *(a)symmetry* of the singularities. The first result on absence of mixing for special flows with *symmetric* logarithmic singularities over *rotations* is due to Kochergin [Ko72] (see also [Ko07] where the result was proved for all irrational frequencies). If the roof has an asymmetric logarithmic singularities, instead, mixing is typical, as it was proved by Sinai-Khanin for a full measure set of rotations numbers (see also further works by Kochergin [Ko75, Ko03, Ko04, Ko04’]). These flows are also known as Arnold flows since Sinai-Khanin result [KS] proved Arnold’s conjecture [Arn] on mixing of typical locally Hamiltonian flows with a saddle point on the torus).

Stronger mixing and spectral properties were later shown for flows over rotations. First, as already mentioned in the introduction, Frączek and Lemańczyk in [FL03] showed that flows under a symmetric logarithm over a full measure set of rotation numbers are disjoint from all mixing flows and have singular spectrum. Fayad and Kanigowski recently proved in [FK] that Arnold flows (as well as some Kochergin flows -i.e. flows under roofs with power-type singularities- over rotations) are mixing of all orders. Kanigowski, Lemańczyk and Ulcigrai proved some disjointness properties (in particular disjointness of rescalings) for typical Arnold flows, [KLU].

**Flows with logarithmic singularities over IETs.** Fewer results are available for flows with logarithmic singularities over IETs. A simple mechanism that shows that weak mixing (or, equivalently, continuity of the spectrum) holds typically as long as there is a logarithmic singularity (recall that weak mixing is also know to hold for typical translation flows by [AF]). Mixing again depends on the symmetry. Scheglov proved in [Sc] that typical minimal locally Hamiltonian flows with isomorphic simple saddles in $g=2$ are not mixing.

Ulcigrai showed in [Ul11] that, for typical IETs, flows with symmetric singularities are not mixing (thus, in the open set $\mathscr {U}_{min}$ of locally Hamiltonian flows, the typical flow, which is minimal and ergodic, is weak mixing but not mixing). Nevertheless, the existence of a mixing flow under a symmetric roof function (smoothly realized by a minimal, locally Hamiltonian flow with only simple saddles on a surface of genus $g=5$) was proved by Chaika and Wright in [CW].

On the other hand, generalizing Sinai-Khanin result [KS], Ulcigrai showed in [Ul07] that flows over IETs with one asymmetric logaritmic singularity are mixing for almost every IET. These result was recently generalized by Ravotti in [Rav] to any number of singularities (thus showing that in presence of saddle loops homologous to zero the typical locally Hamiltonian flow with non-degenerate zeros has mixing minimal components). Recent strengthenings of the mixing property were also proved: Ravotti in [Rav] also proved quantitative (subpolynomial) bounds on the speed of mixing for smooth observables. Finally, in [KKU] it was shown that for a full measure (sub)set of IETs, flows with asymmetric logarithmic singularities are mixing of all orders (and thus that mixing implies mixing of all orders for typical smooth area-preserving flows any genus.)

## A Criterion for Singularity in Special Flows

In this section we present a sufficient condition (originally formulated in [FL03, FL04] in a slightly less general form) which guarantees that $(T^f_t)_{t\in \mathbb {R}}$ has singular spectrum. We first recall some basic spectral theory.

### Spectral notions

The spectrum and the spectral properties of a measure-preserving flow $(T_t)_{t \in \mathbb {R}}$ acting on a probability Borel space $(X,\mathcal {B},\mu )$ are defined in terms of the Koopman (unitary) operators associated to $(T_t)_{t \in \mathbb {R}}$. Let us recall that, for every $t\in \mathbb {R}$, the *Koopman* *operator* associated to the automorphism $T_t$, which, abusing the notation, we will denote also by $T_t$, is the operator

$$\begin{aligned}T_t:L^2(X,\mu )\rightarrow L^2(X,\mu )\quad \text { given by }\ \ T_t(f)=f\circ T_t.\end{aligned}$$

To every $g\in L^2(X,\mu )$ one can associate a spectral measure denoted by $\sigma _g$, i.e. the unique finite Borel measure on $\mathbb {R}$ such that

$$\begin{aligned}\langle g\circ T_{t},g\rangle =\int _\mathbb {R}e^{its}\,d\sigma _g(s)\quad \text {for every}\quad t\in \mathbb {R}.\end{aligned}$$

The spectrum of $(T_t)_{t \in \mathbb {T}}$ is (*purely*) *singular* iff for every $g\in L^2(X,\mu )$ the spectral measure $\sigma _g$ is singular with respect to the Lebesgue measure on $\mathbb {R}$.

Let us denote by $\mathbb {R}(g)\subset L^2(X,\mu )$ the *cyclic subspace* generated by *g* which is given by

$$\begin{aligned}\mathbb {R}(g):={\overline{{\text {span}}}}\{T_t(g):t\in \mathbb {R}\}\subset L^2(X,\mu )\end{aligned}$$

By the spectral theorem (see e.g. [CFS]) the Koopman $\mathbb {R}$-representation $(T^f_{t})_{t\in \mathbb {R}}$ restricted to $\mathbb {R}(g)$ is unitarily isomorphic to the $\mathbb {R}$-representation $(V_t)_{t\in \mathbb {R}}$ on $L^2(\mathbb {R},\sigma _g)$ given by $V_t(h)(s)=e^{its}h(s)$.

Finally, let us recall the notion of *integral operator*. For every probability Borel measure *P* on $\mathbb {R}$ denote by $\int _\mathbb {R}T_t\,dP(t):L^2(X,\mu )\rightarrow L^2(X,\mu )$ the operator such that

$$\begin{aligned} \left\langle \int _\mathbb {R}T_t\,dP(t)(g_1),g_2\right\rangle =\int _\mathbb {R}\langle T_t(g_1),g_2\rangle \,dP(t) \end{aligned}$$

for all $$g_1,g_2\in L^2(X,\mu )$$.

### The singularity criterion

We will now state the singularity criterion for special flows $(T^f_t)$, which are based on rigidity of the base $T:X\rightarrow X$ combined with exponential tails of the Birkhoff sums for the roof function $f:X\rightarrow \mathbb {R}_{>0}$. Let us first recall the notion of *rigidity*.

#### Definition 3.1

*(Rigidity).* An automorphism *T* of a probability Borel space $(X,\mathcal {B},\mu )$ is called *rigid* if there exists an increasing sequence $(h_n)_{n\in \mathbb {N}}$ of natural numbers such that

$$\begin{aligned}\lim _{n\rightarrow +\infty }\mu (A\triangle T^{h_n}A)=0\quad \text {for every}\quad A\in \mathcal {B}.\end{aligned}$$

The sequence $(h_n)_{n\in \mathbb {N}}$ is then a *rigidity sequence* for *T*.

For every $B\in {\mathcal {B}}$ with $\mu (B)>0$, we denote by $\mu _B$ the *conditional measure* given by $\mu _B(A)=\mu (A|B)=\mu (A\cap B)/\mu (B)$ for every measurable *A*.

#### Theorem 3.1

(Singularity Criterion via rigidity and exponential tails). Let $f:X\rightarrow \mathbb {R}_{>0}$ be an integrable roof function with $\inf _{x\in X}f(x)>0$. Suppose that there exist a rigidity sequence $(h_n)_{n\in \mathbb {N}}$ for *T*, a sequence $(C_n)_{n\in \mathbb {N}}$ of Borel sets with $\mu (C_n)\rightarrow 1$ as $n\rightarrow +\infty$, and a sequence of real numbers $(c_n)_{n\in \mathbb {N}}$ (centralizing constants) such that $(S_{h_n}(f)(x)-c_n)_{n\in \mathbb {N}}$ has exponential tails, i.e.  there exists two positive constants *C* and *b* such that

5 $$\begin{aligned} \mu (\{x\in C_n:|S_{h_n}(f)(x)-c_n|\ge t\})\le Ce^{-bt}\text { for all }t\ge 0\text { and }n\in \mathbb {N}. \end{aligned}$$

Then the flow $(T^f_{t})_{t\in \mathbb {R}}$ has singular spectrum.

#### Remark 3.1

The *exponential tails* assumption, i.e. (5), along rigidity sets implies in particular that the sequence of centralized Birkhoff sums $(S_{h_n}(f)(x)-c_n)_{n\in \mathbb {N}}$ is *tight*. Tightness of Birkhoff sums along (partial) rigidity subsequences of the base is at the heart of many criteria for absence of mixing, starting from Katok [Ka80] and Kogergin [Ko72] seminal works. Theorem 3.1 can hence be seen as considerable *strengthening* of this approach to absence of mixing and shows that tightness and rigidity (of the base), with the additional information of exponential tails, is sufficient to show singularity of the spectrum. Contrary to proofs of absence of mixing, though, it is crucial for the spectral conclusion that the rigidity here is *global*, i.e. the measure of the sets $C_n$ tends to 1.

We conclude this section with the proof of the criterion. In the proof we will use the following result from [FL05], which is a version of Prokhorov weak compactness of tight sequences along rigidity sets.

#### Proposition 3.2

(Theorem 6 in [FL05]). Suppose that there exist a rigidity sequence $(h_n)_{n\in \mathbb {N}}$ for *T*, a sequence $(C_n)_{n\in \mathbb {N}}$ of Borel sets with $\mu (C_n)\rightarrow 1$ as $n\rightarrow +\infty$ and a sequence of real numbers $(c_n)_{n\in \mathbb {N}}$ such that

(i) the sequence $(\int _{C_n}|f_n|^2\,d\mu |)_{n\in \mathbb {N}}$ is bounded, where $f_n:=S_{h_n}(f)-c_n$;
(ii) there exists a probability distribution *P* on $\mathbb {R}$ such that $(f_n|{C_n})_*(\mu _{C_n})\rightarrow P$ weakly.

Then, passing to a subsequence if necessary, we have

$$\begin{aligned}T^f_{c_n}\rightarrow \int _\mathbb {R}T^f_{-t}\,dP(t) \text { in the weak operator topology.}\end{aligned}$$

#### Proof of Theorem 3.1

Suppose that, contrary to our claim, the spectral measure $\sigma _g$ (see Sect. 3.1 for the definition) is absolutely continuous for some non-zero $g\in L^2(X^f,\mu ^f)$. Then, by the Riemann-Lebesgue lemma,

6 $$\begin{aligned} V_t\rightarrow 0\text { in the weak operator topology on }L^2(\mathbb {R},\sigma _g)\text { as }|t|\rightarrow +\infty . \end{aligned}$$

Note that, by (5), we have

$$\begin{aligned} \int _{C_n}|S_{h_n}(f)(x)-c_n|^2\,d\mu (x)&\le \sum _{m=0}^\infty \int _{\{x\in C_n:m\le |S_{h_n}(f)(x)-c_n|<m+1\}}(m+1)^2\,d\mu (x)\\&\le \sum _{m=0}^\infty C(m+1)^2e^{-bm}. \end{aligned}$$

Hence, the condition (*i*) in Proposition 3.2 is satisfied. Moreover, also by (5), the sequence of probability Borel measures $((f_n|{C_n})_*(\mu _{C_n}))_{n\in \mathbb {N}}$ on $\mathbb {R}$ is uniformly tight. Therefore, passing to a subsequence, we have $(f_n|{C_n})_*(\mu _{C_n})\rightarrow P$ weakly for some probability measure *P* and the condition (*ii*) in Proposition 3.2 is satisfied. Moreover, *P* has exponentially decaying tails, i.e. there exist $C,b>0$ such that

$$\begin{aligned} P((-\infty ,-t)\cup (t,+\infty ))\le Ce^{-bt}\quad \text {for all}\quad t\ge 0. \end{aligned}$$

In view of Proposition 3.2, we have

$$\begin{aligned} T^f_{c_n}\rightarrow \int _\mathbb {R}T^f_{-t}\,dP(t)\text { in the weak operator topology}. \end{aligned}$$

Restricting this convergence to the invariant subspace $\mathbb {R}(g)$ and passing to $L^2(\mathbb {R},\sigma _g)$, this gives

$$\begin{aligned} V_{c_n}\rightarrow \int _\mathbb {R}V_{-t}\,dP(t)\text { in the weak operator topology on }L^2(\mathbb {R},\sigma _g). \end{aligned}$$

In view of (6), it follows that for all $h_1,h_2\in L^2(\mathbb {R},\sigma _g)$ we have

$$\begin{aligned} 0=&\left\langle \int _\mathbb {R}V_{-t}\,dP(t)(h_1),h_2\right\rangle =\int _{\mathbb {R}}\int _\mathbb {R}e^{-its}h_1(s)\overline{h_2}(s)\,d\sigma _g(s)\,dP(t)\\ =&\int _{\mathbb {R}}{\widehat{P}}(s)h_1(s)\overline{h_2}(s)\,d\sigma _g(s), \end{aligned}$$

where ${\widehat{P}}$ is the Fourier transform of the measure *P*. Therefore (since $h_1$ and $h_2$ are arbitrary), ${\widehat{P}}(s)=0$ for $\sigma _g$ a.e. $s\in \mathbb {R}$. On the other hand, as *P* has exponentially decaying tails, its Fourier transform ${\widehat{P}}$ is an analytic function on $\mathbb {R}$. It follows that ${\widehat{P}}\equiv 0$, contrary to non-triviality of the measure $\sigma _g$. This completes the proof. $\square$

#### Remark 3.2

In fact, the proof of Theorem 3.1 also gives spectral disjointness of $T_f$ from all mixing flows.

## Logarithmic Singularities, Symmetries and Exponential Tails

In this section we will verify that, in the settings of Theorem 1.2 the assumptions of the singularity criterion given by Theorem 3.1 hold.

### Birkhoff sums of functions with logarithmic singularities

In this section we present some estimates on Birkhoff sums of functions with logarithmic singularities which will be used to prove the exponential tails assumption. The results are all elementary, essentially based on the mean value theorem. The precise form of the estimates though allows us to have a detailed control of the behavior of the tails, i.e. the way Birkhoff sums explode due to the presence of singularities.

The following general Lemma shows that control of the exponential tails can be deduced from upper bounds on the second derivative. It will be applied below to $g = S_{h_n}(f)$ (Birhoff sums of *f* along rigidity times $h_n$).

#### Lemma 4.1

(Exponential tails control) Suppose that $g:(a,b)\rightarrow \mathbb {R}$ is a $C^2$-function such that

$$\begin{aligned}|g''(x)|\le \frac{C}{(x-a)^2}+\frac{C}{(b-x)^2}\ \mathrm{for\,every}\,x\in (a,b).\end{aligned}$$

Let $y_0=\tfrac{a+b}{2}$ and assume that there exists $x_0\in (a,b)$ be such that $g'(x_0)=0$. Then for every $x\in (a,b)$ we have

7 $$\begin{aligned} |g(x)-g(y_0)|\le & {} C\Big (-\log \frac{x-a}{b-a}-\log \frac{b-x}{b-a} +\frac{b-a}{x_0-a}+\frac{b-a}{b-x_0}\Big ). \end{aligned}$$

In particular, for every $t\ge 0$ we have

8 $$\begin{aligned} \frac{\lambda (\{x\in (a,b):|g(x)-g(y_0)|\ge t\})}{b-a}\le 2\sqrt{K}e^{-t/2C}, \end{aligned}$$

where $$K:=\tfrac{1}{4}e^{\tfrac{b-a}{x_0-a}+\tfrac{b-a}{b-x_0}}$$.

#### Remark 4.1

Notice that *K* depends only on the point $x_0$ where $g'(x_0)=0$, so that in order for Lemma 4.1 to imply exponential tails estimates for a sequence of functions with a *uniform* constant *K* it is essential to control $x_0$ and in particular its distance from the endpoints *a*, *b*. In Sect. 4.2, Lemma 4.1 will be applied to the function $g=S_{h_n}(f)$ on one of the maximal intervals (*a*, *b*) in which it is continuous. The assumption that there exists $x_0$ such that $S_{h_n}(f')(x_0)=g'(x_0)=0$ follows easily from the fact that $S_{h_n}(f)$ explodes at the endpoints of (*a*, *b*) (and hence has a minimum). On the other hand, the *location* of $x_0 \in (a,b)$ is not in general easy to control and we will crucially use arguments which exploit the hyperelliptic symmetry to control $x_0$.

#### Proof

By assumption, for every $x\in (a,b)$ we have

$$\begin{aligned} |g'(x)|&=|g'(x)-g'(x_0)|\le \Big |\int _{x_0}^x\Big (\frac{C}{(t-a)^2} +\frac{C}{(b-t)^2}\Big )\,dt\Big |\\&\le C\Big (\Big |\frac{1}{b-x}-\frac{1}{x-a}\Big |+\frac{1}{x_0-a}+\frac{1}{b-x_0}\Big ). \end{aligned}$$

It follows that, for every $x\in (a,b)$ we have

$$\begin{aligned} |g(x)-g(y_0)|&\le C\Big |\int _{y_0}^x\Big (\Big |\frac{1}{b-t}-\frac{1}{t-a}\Big |+\frac{1}{x_0-a}+\frac{1}{b-x_0}\Big )\,dt\Big |\\&\le C\Big (\int _{y_0}^x\Big (\frac{1}{b-t}-\frac{1}{t-a}\Big )\,dt+\frac{|x-y_0|}{x_0-a}+\frac{|x-y_0|}{b-x_0}\Big ) \\&\le C\Big (-\log \frac{x-a}{(b-a)/2}-\log \frac{b-x}{(b-a)/2}+\frac{b-a}{x_0-a}+\frac{b-a}{b-x_0}\Big ), \end{aligned}$$

since $|x-y_0|\le b-a$ and recalling that $y_0-a=b-y_0=(b-a)/2$. This gives (7). Moreover, by (7), if $|g(x)-g(y_0)|\ge t$ then

$$\begin{aligned}\frac{(x-a)(b-x)}{(b-a)^2}\le Ke^{-t/C}.\end{aligned}$$

Note also that for any $u>0$ we have

$$\begin{aligned}\frac{\lambda (\{x\in (a,b):\tfrac{(x-a)(b-x)}{(b-a)^2}\le u\})}{b-a}=\lambda (\{x\in (0,1):x(1-x)\le u\})\le 2\sqrt{u},\end{aligned}$$

which gives (8). $$\quad \square $$

The following Lemma (Lemma 4.2) provides an upper bound on the second derivative $S_{h_n}(f'')$ which is exactly of the form needed to verify the assumption of Lemma 4.1 and prove exponential tails.

#### Definition 4.1

(Rokhlin tower by intervals). Let $T:I\rightarrow I$ be an IET with $|I|\le 1$. Given an interval $J_n:=(a_n,b_n)\subset I$ and an integer $h_n\in \mathbb {N}$ we say that the union $\mathcal {C}:=\bigcup _{i=0}^{h_n-1}T^i J_n$ is a (Rokhlin) *tower by intervals* of *base* $J_n$ of *height* $h_n$ if and only if the images $T^i J_n, 0\le i < h_n$ are pairwise disjoint intervals.

We remark that, since each $T^i J_n$ is by assumption an interval (i.e. it was not split by discontinuities of *T*), $J_n$ is an interval of *continuity* for $T^i$ for every $0\le i <h_{n}$.

#### Lemma 4.2

(Second derivative upper bounds). Consider a function $f\in \mathcal {L}\text {og}^\text {p} \left( \sqcup _{i=0}^{d-1}I_i \right)$ of the form

$$\begin{aligned}f(x)=\sum _{0\le i<d}\big (-C_i^+\log (x-\beta _i)-C_{i+1}^-\log (\beta _{i+1}-x)\big )\chi _{(\beta _i,\beta _{i+1})}(x). \end{aligned}$$

Assume that $x\in J_n$ where $J_n:=(a_n,b_n)\subset I$ is the base of a Rokhlin tower by intervals of height $h_n\in \mathbb {N}$. Then

9 $$\begin{aligned} |S_{h_n}(f'')(x)|\le \frac{\pi ^2}{6}\Big (\frac{C^+}{(x-a_n)^2}+\frac{C^-}{(b_n-x)^2}\Big ) \end{aligned}$$

with $C^+=\sum _{i=0}^{d-1}C_i^+$ and $C^-=\sum _{i=1}^dC_i^-$. Moreover, if $a_n<x<x'<b_n$ then for every $0\le h<h_n$, we have

10 $$\begin{aligned} \begin{aligned} |S_{h}(f)(x)-S_{h}(f)(x')|\le&\, C^+\Big (\frac{x'-x}{x-a_n}+\frac{x'-x}{b_n-a_n}\Big (1+\log \frac{1}{b_n-a_n}\Big )\Big )\\&\,+ C^-\Big (\frac{x'-x}{b_n-x'}+\frac{x'-x}{b_n-a_n}\Big (1+\log \frac{1}{b_n-a_n}\Big )\Big ). \end{aligned} \end{aligned}$$

#### Remark 4.2

We stress that (9) holds only for *Birkhoff sums along a tower*, i.e.  a point *x* is in the base of the Rokhlin tower and the Birkhoff sum goes up to height $h_n$ of the tower, while (10) holds for points in the base $J_n$ and for any *intermediate* time $0\le h< h_n$. Under the assumption that *x* is not close to the endpoints $a_n,b_n$ of $J_n$, namely if $x- a_n\ge c(b_n-a_n)$ and $b_n-x \ge c(b_n-a_n)$ for some $0<c<1$, (9) together with Lemma 4.1 provide a uniform bound (independent on *n*) for the difference in (10), while (10) provides only an upper bound of order $C \log (b_n-a_n)^{-1}$. This is a well-known upper bound for functions with *asymmetric* logarithmic singularities (see e.g. [KS, Ul07, Rav], where it is shown that $S_{h}(f')(x)$ grows as $h_n \log h_n$). While Birkhoff sums along a (large) tower are well distributed, incomplete sums can indeed be very unbalanced and only satisfy estimates associated to an asymmetric roof.

#### Proof

Notice first that it is enough to prove (9) and (10) in the special cases when

$$\begin{aligned}&f=f_i^+:=-\log (x-\beta _i)\chi _{(\beta _i,\beta _{i+1})}(x) \quad \text {(and } C^+=1, C^- =0) \text { and}\\&f=f^-_i:=-\log (\beta _{i+1}-x)\chi _{(\beta _i,\beta _{i+1})}(x)\quad \text {(and } C^+=0, C^- =1\text {).} \end{aligned}$$

Indeed, taking the linear combination $\sum _{i=0}^{d-1}C_i^+f_i^+ +C_{i+1}^- f_{i}^-$ then yields the general form of the result. Since the reasoning is analogous for functions of the form $f_i^+$ or $f_i^-$ we will only do the computations for $f=f_i^+$.

For any $x\in J_n$ choose $0\le j<h_n$ such that the iterate $T^jx$ is the closest to $\beta _i$ among all iterates $T^kx$, $0\le k<h_n$ belonging to the interval $(\beta _i, \beta _d)$. Then

$$\begin{aligned}T^jx-\beta _i\ge x-a_n.\end{aligned}$$

Notice that $\inf \{ \vert T^i (x)- T^j(x)\vert , 0 \le i\ne j <h\}\ge b_n-a_n$. Indeed, each point of the orbit $T^h (x)$ for $0\le h < h_n$ belongs to one of the disjoint intervals $\{ T^h J_n, 0\le h < h_n\}$, each of which (since $T^h$ is an isometry on $J_n$) has length $b_n-a_n$. Thus,

$$\begin{aligned} |S_{h_n}(f'')(x)|&\le \sum _{0\le l<h_n}\frac{1}{(T^jx-\beta _i+l(b_n-a_n))^2} \le \frac{1}{(x-a_n)^2}\sum _{0\le l<h_n}\frac{1}{(1+l\tfrac{b_n-a_n}{x-a_n})^2}\\&\le \frac{1}{(x-a_n)^2}\sum _{l\ge 1}\frac{1}{l^2}=\frac{\pi ^2}{6}\frac{1}{(x-a_n)^2}. \end{aligned}$$

This gives (9).

Suppose now that $a_n<x<x'<b_n$ and $f=f_i^+$. Let $\delta :=x'-x$ and $\varepsilon :=x-a_n$. Then for any $0\le h<h_n$ (noticing that since the area of a tower is less than one, $h_n(b_n-a_n) \le 1$),

$$\begin{aligned} |S_h(f)(x')-S_h(f)(x)|&\le \sum _{\begin{array}{c} 0\le k<h_n\\ T^kx>\beta _i \end{array}}\log \frac{(T^kx-\beta _i)+\delta }{T^kx-\beta _i}\le \sum _{\begin{array}{c} 0\le k<h_n\\ T^kx>\beta _i \end{array}}\frac{\delta }{T^kx-\beta _i}\\&\le \sum _{0\le l<h_n}\frac{\delta }{(T^jx-\beta _i)+l(b_n-a_n)} \le \sum _{0\le l<h_n}\frac{\delta }{\varepsilon +l(b_n-a_n)}\\&\le \frac{\delta }{\varepsilon }+\sum _{1\le l<h_n}\frac{\delta }{l(b_n-a_n)} \le \frac{\delta }{\varepsilon }+\frac{\delta }{b_n-a_n}(1+\log h_n)\\&\le \frac{\delta }{\varepsilon }+\frac{\delta }{b_n-a_n}\Big (1+\log \frac{1}{b_n-a_n}\Big ). \end{aligned}$$

In virtue of the initial remark, this concludes the proof of (10). $\quad \square$

From Lemmas 4.1 and 4.2, we can deduce exponential tails as long as we can control the location of a zero of $S_{h_n}(f')$.

#### Corollary 4.3

Let $y_n=\tfrac{a_n+b_n}{2}$. Assume that there exists $0<c<1/2$ such that for every $n\ge 1$ we have $S_{h_n}(f')(x_n)=0$ for some $x_n\in [a_n+c(b_n-a_n),b_n-c(b_n-a_n)]$. Then for every $n\ge 1$ and $t\ge 0$ we have

$$\begin{aligned} \frac{1}{b_n-a_n}\lambda (\{x\in J_n:|S_{h_n}(f)(x)-S_{h_n}(f)(y_n)|\ge t\})\le e^{1/c}e^{-t/2C}, \end{aligned}$$

where $$C=\frac{\pi ^2}{6}\max \left\{ \sum _{i=0}^{d-1}C_i^+,\sum _{i=1}^dC_i^-\right\} $$.

#### Proof

This is a consequence of Lemma 4.1 (applied to $g=S_{h_n}(f)$) and Lemma 4.2: it is enough to notice that since $x_n\in [a_n+c(b_n-a_n),b_n-c(b_n-a_n)]$, the constant *K* (see Lemma 4.1) is globally bounded (in terms of $c>0$). Indeed,

$$\begin{aligned}K=\tfrac{1}{4}e^{\tfrac{b_n-a_n}{x_n-a_n}+\tfrac{b_n-a_n}{b_n-x_n}} \le \frac{e^{\tfrac{2}{c}}}{4},\end{aligned}$$

which completes the proof. $\quad \square$

### Hyperelliptic symmetry and cancellations

In this section we show that the symmetries of an IET with a symmetric $\pi$ and a roof function with pure symmetric logarithmic singularities allow us to determine critical points of $S_{h_n} (f)$.

#### Remark 4.3

Let $(T_t)_{t\in \mathbb {R}}$ be a measure-preserving flow on $(X,\mathcal {B},\mu )$ and let *S* be a measure-preserving involution such that

11 $$\begin{aligned} T_t\circ S=S\circ T_{-t}\;\text { for every }\;t\in \mathbb {R}. \end{aligned}$$

Let $I\subset X$ be a global transversal for the flow $(T_t)_{t\in \mathbb {R}}$ such that $S(I)=I$. Let $T_I:I\rightarrow I$ be the first return map to *I* and $f:I\rightarrow \mathbb {R}_{>0}$ be the first return time. Then, it is easy to check that,

12 $$\begin{aligned} T_I\circ S=S\circ T_I^{-1}\;\text { and }\;f\circ T_I^{-1}\circ S=f. \end{aligned}$$

In fact, the conditions (11) and (12) are in a sense equivalent. Indeed, if $S:I\rightarrow I$ is an involution satisfying (12), then it has an extension to the involution $S^f:I^f\rightarrow I^f$ given by

$$\begin{aligned}S^f(x,y):=(Sx,-y)\;\text { for all }\;(x,y)\in I^f.\end{aligned}$$

Then (12) implies (11) for the special flow $T_I^f$.

Assume throughout this section that $T:I\rightarrow I$ is an IET associated with the *symmetric* permutation $\pi (i)=d-1-i$ for $0\le i < d$. Recall that $\mathcal {S}\text {ym}\mathcal {L}\text {og}^\text {p} \left( \sqcup _{i=0}^{d-1}I_i \right)$ denotes functions with *pure symmetric logarithmic singularities* (see Definition 2.3). Such IETs and functions enjoy the following symmetries.

#### Lemma 4.4

(Symmetries) Let *T* be an IET with a symmetric $\pi$ and endpoints $0=\beta _0< \dots <\beta _d=|I|$ and assume $f \in \mathcal {S}\text {ym}\mathcal {L}\text {og}^\text {p} \left( \sqcup _{i=0}^{d-1}I_i \right)$. Then, if $S:I\rightarrow I$ denotes the involution $S(x)=|I|-x$,

(SB) $$T\circ S=S\circ T^{-1}$$;
(SR) $$f'\circ T^{-1}\circ S=-f'$$.

[*SB* stands for Symmetries of the Base and *SR* for Symmetries of the Roof.] Notice that the relation (*SB*) is the same that appeared in the proof of Lemma 2.1, see (4).

#### Proof

Since $\pi$ is symmetric, *T* maps $[\beta _i,\beta _{i+1})$ linearly on $[|I|-\beta _{i+1},|I|-\beta _{i})$, i.e.

$$\begin{aligned}Tx=x+|I|-\beta _i-\beta _{i+1}\text { for all }x\in [\beta _i,\beta _{i+1}).\end{aligned}$$

Thus, one can verify directly that (*SB*) holds.

We claim that a measurable function $\phi :I\rightarrow \mathbb {R}\cup \{\pm \infty \}$ satisfies $\phi \circ T^{-1}\circ S=-\phi$ if and only if

13 $$\begin{aligned} \phi (x)=-\phi (\beta _i+\beta _{i+1}-x)\text { for all }x\in (\beta _i,\beta _{i+1})\text { and any }0\le i< d. \end{aligned}$$

Indeed, for every $x\in (\beta _i,\beta _{i+1})$ we have

$$\begin{aligned}\phi ( T^{-1} (Sx))=\phi ( T^{-1} (|I|-x))=\phi (|I|-x -(|I|-\beta _i-\beta _{i+1}))=\phi (\beta _i+\beta _{i+1}-x).\end{aligned}$$

This gives our claim.

Since $$f \in \mathcal {S}\text {ym}\mathcal {L}\text {og}^\text {p} \left( \sqcup _{i=0}^{d-1}I_i \right) $$,

14 $$\begin{aligned} f'(x)=-\frac{C^+_i}{x-\beta _i}+\frac{C^-_{i+1}}{\beta _{i+1}-x}\text { if }x\in (\beta _i,\beta _{i+1})\qquad \text { for }0\le i< d, \end{aligned}$$

where $C^+_i=C^-_{i+1}=C$ for $0\le i< d$. Hence one sees that $\phi := f'$ satisfies (13) and hence (*SR*) holds. $\quad \square$

#### Remark 4.4

By the proof of Lemma 4.4, we also have that for every $f \in \mathcal {L}\text {og}^\text {p} \left( \sqcup _{i=0}^{d-1}I_i \right)$ the symmetry condition ($C^+_i=C^-_{i+1}$ for $0\le i< d$) is equivalent to $f\circ T^{-1}\circ S=f$.

The relations in Lemma 4.4 automatically imply the symmetry of Birkhoff sums stated in Lemma 4.5 below and hence allows us to locate $x_0$ such that $S_n( f')(x_0)=0$ (see Corollary 4.6).

#### Lemma 4.5

(Cancellations). Suppose that *T* and *S* are measure-preserving automorphisms of a probability Borel space $(X,\mathcal {B},\mu )$ such that $T\circ S=S\circ T^{-1}$ and *S* is idempotent ($S^2=Id$). Assume that $\phi :X\rightarrow \mathbb {R}$ is a measurable map with $\phi \circ T^{-1}\circ S=-\phi$. Then for every $n\in \mathbb {N}$ and $x\in X$ we have

$$\begin{aligned} S_n(\phi )(T^{-n}(Sx))=-S_n(\phi )(x). \end{aligned}$$

In particular, if $x_0\in X$ is a fixed point of *S* ($Sx_0=x_0$), then for every $n\in \mathbb {N}$ we have

$$\begin{aligned} S_{n}(\phi )(T^{-n}x_0)=-S_{n}(\phi )(x_0). \end{aligned}$$

#### Proof

The first part follows simply by the chain of equalities

$$\begin{aligned} S_n(\phi )(T^{-n}(Sx))&=\sum _{0\le i<n}\phi (T^{i-n}(Sx))=\sum _{0\le i<n}\phi (T^{-1-i}(Sx))\\&=\sum _{0\le i<n}\phi (T^{-1}(S(T^ix))) =-\sum _{0\le i<n}\phi (T^ix)=-S_n(\phi )(x). \end{aligned}$$

The second part is also immediate. $\quad \square$

Combining Lemmas 4.4 and 4.5 we have the following Corollary.

#### Corollary 4.6

(Cancellations). Let *T* be an IET with a symmetric $\pi$ and endpoints $0=\beta _0< \dots <\beta _d=|I|$ and $f \in \mathcal {S}\text {ym}\mathcal {L}\text {og}^\text {p} \left( \sqcup _{i=0}^{d-1}I_i \right)$. For every $n\in \mathbb {N}$, we have

15 $$\begin{aligned} S_n(f')(T^{-n}x_0)=-S_n(f')(x_0)\ \ \text {for} \ \ x_0=|I|/2. \end{aligned}$$

Moreover, if (*a*, *b*) is an interval on which $S_{n}(f')$ is continuous and both $x_0$ and $T^{-n}x_0$ belong to (*a*, *b*), it follows that there exists

$$\begin{aligned} x_n \in (a,b)\quad \text {such that}\quad S_{n}(f')(x_n)=0. \end{aligned}$$

#### Proof

By Lemma 4.4, the assumptions of Lemma 4.5 hold for $T: I \rightarrow I$, $f': I \rightarrow \mathbb {R}$ and $S:I \rightarrow I$ given by $S(x)=|I|-x$. Since $x_0=|I|/2$ is the (unique) fixed point of the involution *S*, the first part follows immediately from Lemma 4.5.

We claim that the second part is simply an application of the intermediate value theorem. Indeed, first note that since $S_{n}(f')$ is by assumption continuous on (*a*, *b*) and *f* has pure logarithmic singularities, it is actually smooth. By the first part of the Corollary, $S_{n}(f')(T^{-n}x_0)=-S_{n}(f')(x_0)$ and by assumption both $x_0$ and $T^{-n}x_0$ belong to (*a*, *b*), so $S_n(f')$ changes sign on (*a*, *b*) and hence must have a zero. $\quad \square$

### Good rigidity and exponential tails

The last ingredient we need to verify the assumptions of the singularity criterion are rigidity sequences given by Rokhlin towers with good recurrence on the base (in the sense of Definition 4.2 below). Recall that Rokhlin towers by intervals were defined at the beginning of Sect. 4.1 (see Definition 4.1).

#### Definition 4.2

(Good rigidity). We say that $T: I\rightarrow I$ admits a *good rigidity sequence* if there exists a sequence of Rokhlin towers by intervals $\mathcal {C}_n\subset I$ of base $J_n=[a_n,b_n]$ and height $h_n$ such that

1. $$\lambda (\mathcal {C}_n)\rightarrow |I|$$

and, if we define $q_n =\frac{1}{b_n-a_n}$ and $\varepsilon _n:= \frac{1}{q_n\log q_n}$,

(GR2) the tower $\mathcal {C}_n$ is $\varepsilon _n$-*rigid*, that is, for every $x\in \mathcal {C}_n$, we have
  $$\begin{aligned} |T^{h_n}x-x|\le \varepsilon _n:= \frac{1}{q_n\log q_n}. \end{aligned}$$

This *good* form of *recurrence* (which will be deduced in Sect. 4.4 by the abundance of directions well approximated by cylinders, see Lemma 4.10) provides the final key ingredient to the proof of singularity of the spectrum for special flows with symmetric logarithmic singularities.

#### Proposition 4.7

(Singularity for symmetric logarithmic flows from good rigidity). Let *T* be an IET with a symmetric permutation $\pi$ and endpoints $0=\beta _0< \dots < \beta _d=|I|$ and assume that $f \in \mathcal {S}\text {ym}\mathcal {L}\text {og} \left( \sqcup _{0\le i<d} I_i \right)$ has symmetric logarithmic singularities.

If *T* admits a *good rigidity sequence* of Rokhlin towers $\mathcal {C}_n\subset I$ with bases $J_n=[a_n,b_n]$ and heights $h_n$ such that, for some $0<c<1/2$, for every $n \in \mathbb {N}$ there exists a point $x_n \in J_n$ such that

16 $$\begin{aligned} x_n\in [a_n+c/q_n,b_n-c/q_n] \quad \text {and} \quad S_{h_n} f'(x_n)=0, \end{aligned}$$

then the special flow $(T_t^f)_{t\in \mathbb {R}}$ is ergodic and has purely singular spectrum.

The proof is given below, using the following two Lemmas.

#### Lemma 4.8

(Exponential tails). Suppose that $f \in \mathcal {S}\text {ym}\mathcal {L}\text {og}^\text {p} \left( \sqcup _{0\le i<d} I_i \right)$. Under the same assumptions as in Proposition 4.7, if $\mathcal {C}'_n$ is a subtower of $\mathcal {C}_n$ of height $h_n$ and whose base is $J'_n=[a_n+2\varepsilon _n,b_n-2\varepsilon _n]$, then there exists $B>0$ such that for every $n\ge 1$ and $t\ge B$ we have

$$\begin{aligned} \lambda (\{x\in \mathcal {C}'_n:|S_{h_n}(f)(x)-S_{h_n}(f)(y_n)|\ge t\})\le |I|e^{1/c}e^{-(t-B)/2C}, \end{aligned}$$

where

$$\begin{aligned} C:=\frac{\pi ^2}{6}\sum _{i=0}^{d-1}C_i^+=\frac{\pi ^2}{6}\sum _{i=1}^dC_i^-, \qquad y_n=\frac{a_n+b_n}{2}.\end{aligned}$$

#### Proof

*Step 1* (*x* *in the base).* Assume first that $x\in \mathcal {C}'_n$ belongs to $J'_n$. Since by assumption there exists $x_n\in [a_n+c/q_n,b_n-c/q_n]$ such that $S_{h_n}(f')(x_n)=0$ we can apply Corollary 4.3 which shows that

17 $$\begin{aligned} q_n\lambda (\{x\in J'_n:|S_{h_n}(f)(x)-S_{h_n}(f)(y_n)|\ge t\})\le e^{1/c}e^{-t/2C}. \end{aligned}$$

*Step 2 (comparing* $y \in J_n'$ *and* $x=T^hy$ *for* $0\le h<h_n$). Consider now any $x\in \mathcal {C}'_n$ and write it as $x=T^hy$ for some $y\in [a_n+2\varepsilon _n,b_n-2\varepsilon _n]$ and $0\le h<h_n$. Then

$$\begin{aligned} |S_{h_n}(f)(x)-S_{h_n}(f)(y)|=|S_{h}(f)(y)-S_{h}(f)(T^{h_n}y)| \end{aligned}$$

with

$$\begin{aligned} |y-T^{h_n}y|\le \varepsilon _n\;\text { and }\;y,T^{h_n}y\in [a_n+\varepsilon _n,b_n-\varepsilon _n]. \end{aligned}$$

Hence, by (10),

$$\begin{aligned} |S_h(f)(y)-S_h(f)(T^{h_n}y)|&\le 2C\big (1+\varepsilon _nq_n(1+\log q_n)\big )\le 2C\Big (1+\frac{1+\log q_n}{\log q_n}\Big )\\&\le 6C=:B. \end{aligned}$$

Therefore, $$|S_{h_n}(f)(x)-S_{h_n}(f)(y)|\le B$$.

*Step 3 (general case).* By the triangle inequality, adding and subtracting $S_{h_n}(f)(y)$, where *y* is chosen so that $x=T^hy$ as in *Step 2*, we have that $|S_{h_n}(f)(x)-S_{h_n}(f)(y_n)|\ge t$ implies $|S_{h_n}(f)(y)-S_{h_n}(f)(y_n)|\ge t-B$. In view of (17), it follows that

$$\begin{aligned} \lambda (\{x\in \mathcal {C}'_n:|S_{h_n}(f)(x)-S_{h_n}(f)(y_n)|\ge t\})\le \frac{h_n}{q_n}e^{1/c}e^{-(t-B)/2C}\le |I|e^{1/c}e^{-(t-B)/2C}. \end{aligned}$$

This concludes the proof. $\quad \square$

Since any function $f\in \mathcal {S}\text {ym}\mathcal {L}\text {og} \left( \sqcup _{i=0}^{d-1}I_i \right)$ by definition can be written as $f=f_p+g$, where $f_p\in \mathcal {S}\text {ym}\mathcal {L}\text {og}^\text {p} \left( \sqcup _{i=0}^{d-1}I_i \right)$ and *g* is a function of bounded variation, the last element we need to prove Proposition 4.7 is to control Birkhoff sums of *g*, through the following standard Denjoy-Koksma-type Lemma.

#### Lemma 4.9

(Estimate for bounded variation functions). Let $g:I\rightarrow \mathbb {R}$ be a function of bounded variation equal to $V\ge 0$. Then for all $x\in \mathcal {C}'_n$ and $x'\in J'_n$ we have

$$\begin{aligned}|S_{h_n}(g)(x)-S_{h_n}(g)(x')|\le 2V.\end{aligned}$$

#### Proof

First note that if $x,x'\in J_n$ and $0\le h<h_n$ then

$$\begin{aligned}|S_{h}(g)(x)-S_{h}(g)(x')|\le V.\end{aligned}$$

Indeed, since

$$\begin{aligned}|S_{h}(g)(x)-S_{h}(g)(x')|\le \sum _{0\le j<h}|g(T^jx)-g(T^jx')|\end{aligned}$$

and the intervals $[T^jx,T^jx']$ for $0\le j<h$ are pairwise disjoint, the right sum is bounded from above by the variation of *g*.

If $x\in \mathcal {C}'_n$ then $x=T^hy$ for some $y\in [a_n+2\varepsilon _n,b_n-2\varepsilon _n]$ and $0\le h<h_n$. Then

$$\begin{aligned} |S_{h_n}(f)(x)-S_{h_n}(f)(y)|=|S_{h}(f)(y)-S_{h}(f)(T^{h_n}y)| \end{aligned}$$

with $|y-T^{h_n}y|\le \varepsilon _n$ and $y,T^{h_n}y\in J_n$. It follows that

$$\begin{aligned} |S_{h_n}(f)(x)-S_{h_n}(f)(x')|\le & {} |S_{h}(f)(y)-S_{h}(f)(T^{h_n}y)|\\&+|S_{h_n}(f)(y)-S_{h_n}(f)(x')|\le 2V. \end{aligned}$$

$$\square $$

We can now use Lemmas 4.8 and 4.9 to prove Proposition 4.7.

#### Proof of Proposition 4.7

We will verify the assumptions of the singularity criterion (Theorem 3.1). Let us remark first that, by assumption, *T* is a rank 1 transformation and hence it is ergodic, see [Fe, Theorem 2]. To check the exponential tails assumption, recall first that, by definition, $f\in \mathcal {S}\text {ym}\mathcal {L}\text {og} \left( \sqcup _{i=0}^{d-1}I_i \right)$ (see Def. 2.3 ) can be written as $f=f_p+g$ where $f_p\in \mathcal {S}\text {ym}\mathcal {L}\text {og}^\text {p} \left( \sqcup _{i=0}^{d-1}I_i \right)$ and *g* has bounded variation. Thus, if *V* denotes the total variation of *g*, by Lemma 4.9,

$$\begin{aligned} |S_{h_n}(f)(x)-S_{h_n}(f)(y_n)|\le |S_{h_n}(f_p)(x)-S_{h_n}(f_p)(y_n)|+2 V\;\text { for every}\; x\in \mathcal {C}'_n \end{aligned}$$

and, $|S_{h_n}(f)(x)-S_{h_n}(f)(y_n)|\ge t$ implies $|S_{h_n}(f_p)(x)-S_{h_n}(f_p)(y_n)|\ge t-2V$. Thus, by Lemma 4.8 applied to the function $f_p$, for every $n\ge 1$ and $t\ge B+2V$ we have

$$\begin{aligned} \lambda (\{x\in \mathcal {C}'_n:|S_{h_n}(f)(x)-S_{h_n}(f)(y_n)|\ge t\})\le |I|e^{1/c}e^{-(t-B-2V)/2C}, \end{aligned}$$

with $\lambda (\mathcal {C}'_n)\rightarrow 1$. Since by assumption $(h_n)$ is a rigidity sequence and the previous equation gives the exponential tails assumption (5), the singularity criterion given by Theorem 3.1 implies that the special flow $(T_t^f)_{t \in \mathbb {R}}$ has singular spectrum. $\quad \square$

### Final arguments

We will now show how to conclude the proof of Theorems 1.1 and 1.2. We will use Proposition 4.7 (which we just proved) and Propostion 1.3, which we will prove in the next and final section. To prove Theorem 1.1, we also need the following Lemma, which relates the notion of *good rigidity* (see Definition 4.2) to the conclusion of Proposition 1.3.

#### Lemma 4.10

(Good rigidity from cylinders). Let $(h_t)_{t\in \mathbb {R}}$ be the vertical translation flow on an area one translation surface $(M, \omega )$. Assume that there exists a sequence $(C_n)_{n \in \mathbb {N}}$ of cylinders with $a({C_n})\rightarrow 1$ and $\ell ({C_n})\rightarrow +\infty$ as $n\rightarrow +\infty$ such that

18 $$\begin{aligned} \vert \theta _{C_n}-\tfrac{\pi }{2}\vert <\frac{1}{\ell ({C_n})^2 \log (\ell ({C_n}))}. \end{aligned}$$

Let $I\subset M$ be a horizontal interval such that both of its endpoints lie on a separatrix and are the first meeting point (forward or backward) of the separatrix and *I*. If $T: I \rightarrow I$ is an IET obtained as the Poincaré map of $(h_t)_{t\in \mathbb {R}}$, then *T* admits a good rigidity sequence.

The idea behind Lemma 4.10 is simply that towers for the IET can be essentially obtained intersecting the cylinders $C_n$ with the Poincaré section. Before we prove Lemma 4.10, we make the following remark which simplifies the analysis.

#### Remark 4.5

We will without loss of generality assume that the endpoints of $I=[a,b]$ do not belong to ${C}_n$ for every $n\in \mathbb {N}$. Indeed, assume that $a\in C_n$. By definition of *I*, $a=h_{-{s}}(\sigma )$, for a singularity $\sigma \in M$ and $|{s}|<C$ (where $C>0$ is a constant independent on *n*, chosen to be an upper bound for backward and forward first return times of singularities to the section). Since $h_{s}(a)=\sigma \notin C_n$, we find ${s'}$ between 0 and *s* such that $h_{{s}'}(a)\in \partial C_n$ and $|{s}'|$ is the smallest positive real number with such property. Choose $v\in \partial C_n$ so that the triangle with vertices $h_{{s}'}(a)$, *v*, *a* is right (*v* is its right angle vertex) and contained in $\overline{C_n}$. Then $\tfrac{d(a,v)}{|{s}'|}=|\sin (\pi /2-\theta _{C_n})|$. It follows that

$$\begin{aligned} d(a,\partial C_n)\le d(a,v)=|{s}'||\sin (\tfrac{\pi }{2}-\theta _{C_n})|\le |{s}||\tfrac{\pi }{2}-\theta _{C_n}|\le \frac{C}{\ell (C_n)^2\log (\ell (C_n))}. \end{aligned}$$

Analogous estimates hold for the other endpoint. If we define *trimmed* cylinders $(C'_n)_{n\in \mathbb {N}}$ given by:

$$\begin{aligned} C'_n:=C_n\setminus \Big \{x\in C_n\;:\; d(x,\partial C_n)\le \frac{C}{\ell (C_n)^2\log (\ell (C_n))}\Big \}, \end{aligned}$$

then the discarded set has measure at most $\frac{2C}{\ell (C_n)\log (\ell (C_n))}$ and therefore we also have that $a(C'_n)\rightarrow 1$ as *n* grows. Since $\theta _{C'_n}=\theta _{C_n}$ and $\ell (C'_n)=\ell (C_n)$, the sequence of cylinders $(C'_n)_{n \in \mathbb {N}}$ also satisfies (18). Therefore we can replace the sequence $(C_n)_{n\in \mathbb {N}}$ with the sequence of cylinders $(C'_n)_{n\in \mathbb {N}}$, which by construction do not contain the endpoints of *I*.

With the above remark we can now prove Lemma 4.10.

#### Proof of Lemma 4.10

Let $(C_n)_{n \in \mathbb {N}}$ be the sequence of cylinders satisfying the assumption of Lemma 4.10 and let *I* denote, by abusing the notation, also the horizontal interval on *M* which gives the Poincaré section determining *T*. Let $({\overline{a}}_n,{\overline{b}}_n)\subset I$ be one of connected components of the intersection of $C_n$ with *I* and set $h_n$ to be the number of connected components in $I \cap C_n$. In view of Remark 4.5, all these connected components are horizontal intervals of length ${\overline{b}}_n-{\overline{a}}_n$ whose endpoints both lie on $\partial C_n$. The images of the intervals $({\overline{a}}_n,{\overline{b}}_n)$ under the vertical flow (and hence the successive intersections of $C_n$ with *I*) are not necessarily disjoint, but since the vertical flow is close by (18) to the direction $\theta _{C_n}$ of the cylinder, to obtain the base of a tower it is sufficient to *trim* the interval as follows. Let $J_n:=({a}_n,{b}_n)$ to be the smaller interval given by

$$\begin{aligned}a_n:= {\overline{a}}_n+ \epsilon _n, \qquad b_n:= {\overline{b}}_n- \epsilon _n ,\qquad \text {where} \ \ \epsilon _n:= \frac{1}{\ell ({C_n}) \log (\ell ({C_n}))}. \end{aligned}$$

**Fig. 4:**
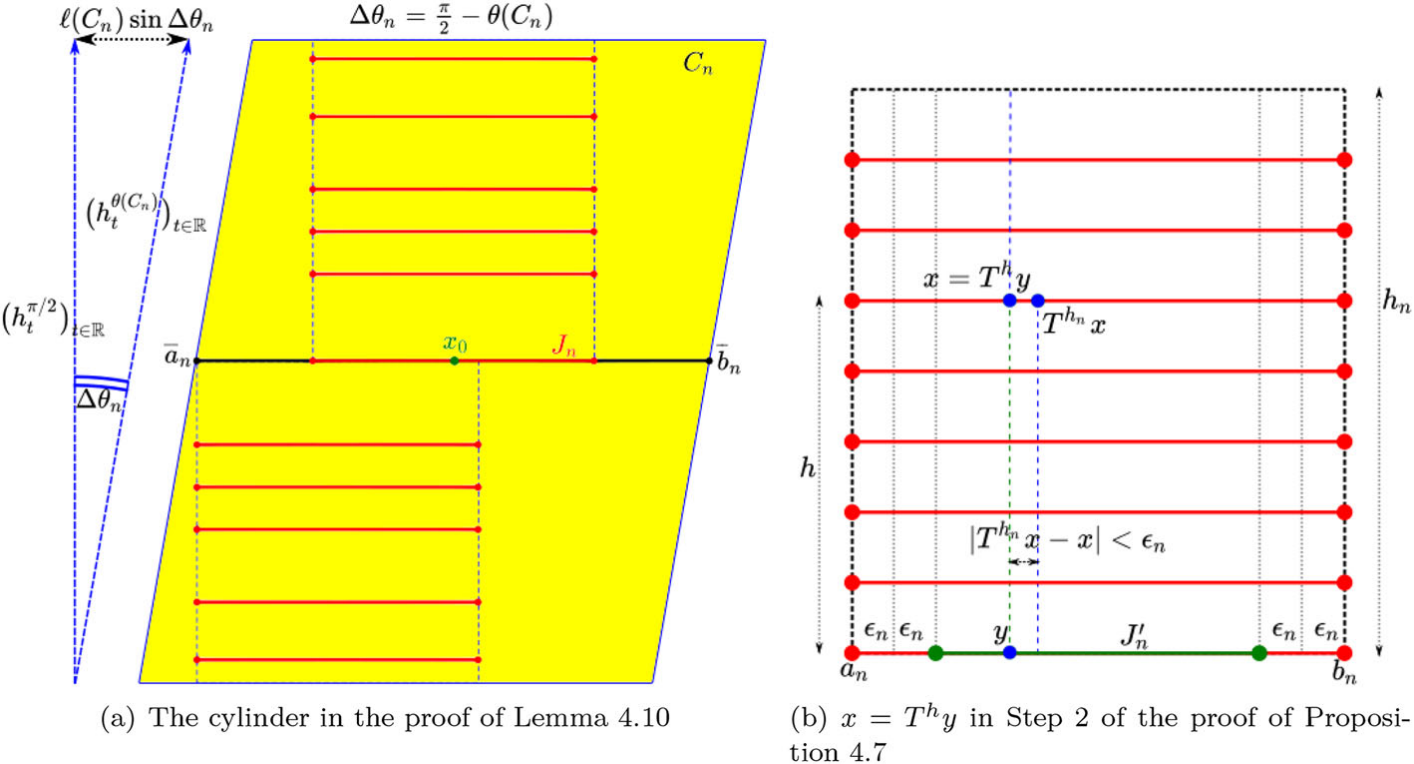
Auxiliary figures for the proofs of Lemma 4.10 and Proposition 4.7

Then, by (18) and elementary trigonometry (see Fig. 4a), we have that the symmetric difference of $T^{h_n}J_n$ and $J_n$ has length

$$\begin{aligned} \ell ({C_n}) \big |\sin \left( \theta _{C_n}-\tfrac{\pi }{2} \right) \big |\le \ell ({C_n}) \vert \theta _{C_n}-\tfrac{\pi }{2}\vert \le \frac{1}{\ell ({C_n}) \log (\ell ({C_n}))} =\epsilon _n. \end{aligned}$$

Note first that by Remark 4.5 it follows that the sets $T^h(J_n)$ for $0\le h <h_n$ are intervals. Moreover, they are pairwise disjoint and we also have that $\vert T^{h_n}(x) - x \vert \le \epsilon _n$ for any *x* from these intervals. Setting $(\mathcal {C}_n)_{n \in \mathbb {N}}$ to be given by $\mathcal {C}_n:=\cup _{h=0}^{h_n-1}T^h (J_n)$, we obtain towers which satisfy (*GR*2) from the Definition 4.2. To finish the proof it is enough to show that the towers $(\mathcal {C}_n)_{n \in \mathbb {N}}$ satisfy (*GR*1). Consider the subsurface $F_n\subset M$ (obtained flowing $J_n$) given by $F_n:=\bigcup _{0\le t<\sin (\theta _{C_n})\ell (C_n)}h_t(J_n)$ (the flowing time $\sin (\theta _{C_n})\ell (C_n)$ is here the smallest first return time of points in $J_n$ to $J_n$, cf. Fig. 4). Denote by $p_I:M\rightarrow I$ the projection along the vertical flow of *M* on *I* defined by setting $p_I(x)$ to be the first meeting point of the backward orbit of *x* under $(h_t)_{t\in \mathbb {R}}$ with *I*. Then $\mathcal {C}_n=p_I(F_n)$. Moreover, by the bound on $\theta _{C_n}$,

$$\begin{aligned} a(F_n)&=|J_n|\cdot \ell (C_n)\sin (\theta _{C_n})=({\bar{b}}_n-{\bar{a}}_n-2\epsilon _n)\ell (C_n)\sin (\theta _{C_n})\\&= ({\bar{b}}_n-{\bar{a}}_n)\ell (C_n)\sin (\theta _{C_n})- 2\epsilon _n\ell (C_n)\sin (\theta _{C_n})\ge a(C_n)-\frac{2}{\log (\ell (C_n))}\rightarrow 1. \end{aligned}$$

Then, if $c>0$ denotes the minimum of all backward first return times of points from *I* to *I* and recall that $|\cdot |$ denotes the Lebesgue measure of a (measurable) subset of *I*, we have

$$\begin{aligned} c\left| p_I(M\setminus F_n)\right| \le a(M\setminus F_n)\rightarrow 0. \end{aligned}$$

Therefore $$\left| p_I(M\setminus F_n)\right| \rightarrow 0$$. Consequently

$$\begin{aligned} |\mathcal {C}_n|=|p_I(F_n)|=|I|-\left| p_I(M\setminus F_n)\right| \rightarrow |I|. \end{aligned}$$

This finishes the proof of (*GR*1). $\quad \square$

We can now prove Theorem 1.2. Let us first outline the strategy of the proof. Singularity of the spectrum for special flows satisfying the assumptions of Theorem 1.2 will be deduced from an application of Proposition 4.7. Hence we only need to verify that the assumptions (good rigidity and location of zeros of the derivatives) hold for almost every *T* with permutation $\pi$. In *Part 1*, *good rigidity* is deduced from Proposition 1.3 via Lemma 4.10. In *Part 2*, the assumption (16) on the *location of zeros* of the derivative is proven exploiting the symmetry of the function and the cancellation phenomena described in Sect. 4.2 (via Corollary 4.6).

#### Proof of Theorem 1.2

*Part 1.* Let $d\ge 2$ and consider the symmetric permutation $\pi$ on *d* symbols. Consider translation surfaces (for example obtained by choosing suspension data, see [Yo]) which has an IET *T*, with permutation $\pi$ as a Poincaré section. Any such translation surface $(M, \omega )$ belongs to the stratum $\mathcal {H}=\mathcal {H}(2g-2)$ where $g=d/2$ if *d* is even, or to $\mathcal {H}=\mathcal {H}(g-1,g-1)$ where $g=(d-1)/2$ if *d* is odd. By Proposition 1.3, for almost every translation surface in (any connected component of) the stratum $\mathcal {H}(2g-2)$ or $\mathcal {H}(g-1,g-1)$, the vertical flow is well approximated by single cylinders in the sense of Proposition 1.3. Furthermore, since Proposition 1.3 holds for every $\epsilon >0$, taking a sequence $\epsilon _n\rightarrow 0$ and using a diagonal argument, we also have that for a full measure set $\mathscr {F}_\mathcal {H}$ of translation surface in either strata there exists a sequence of cylinders $(C_n)_{n\in \mathbb {N}}$ with $a(C_n)\rightarrow 1$ and satisfying (18). We can also assume that for every surface $(M,\omega )$ in $\mathscr {F}_\mathcal {H}$ the corresponding horizontal flow has no saddle connection.

By standard arguments (using Fubini theorem and the local product structure of the Masur-Veech measure on translation surfaces), we hence get a full measure set $\mathscr {F}_{\pi }$ of IETs with permutation $\pi$ (those which arise as Poincaré sections of the vertical flow on surfaces in $\mathscr {F}_\mathcal {H}$) that, by Lemma 4.10, admit a good rigidity sequence (in the sense of Definition 4.2).

*Part 2.* Given $T\in \mathscr {F}_{\pi }$, let $(M, \omega )$ be a translation surface in $\mathscr {F}_\mathcal {H}$ of which *I* is a horizontal section. Let $\iota : M \rightarrow M$ denote the hyperelliptic involution on $(M, \omega )$ (see Sect. 2.7). Since $\pi$ is symmetric, the midpoint $x_0=|I|/2$ of *T* is fixed by $\iota$ (i.e. is a Weierstrass point). Let $(C_n)_{n\in \mathbb {N}}$ be the sequence of cylinders on $(M,\omega )$ satisfying (18) and $a({C_n})\rightarrow 1$. We can assume that each cylinder $C_n$ is maximal. Since, for *n* sufficiently large, $a({C_n})>1/2$, we must have $\iota (C_n) = C_n$. Indeed, since $\iota$ maps cylinders into cylinders and preserves area, $\iota (C_n)$ is a cylinder intersecting $C_n$ (as $a(\iota (C_n))>1/2$ and $a(C_n)>1/2$). Therefore $C_n\cup \iota (C_n)$ is also a cylinder. By the maximality of $C_n$, it follows that $\iota (C_n) = C_n$. This implies that there is a Weierstrass point (actually exactly two) on the core curve of $C_n$. We claim that, without loss of generality, we can assume that for all $n \in \mathbb {N}$ this Weierstrass point is the mid-point $x_0$ of *I*. (Indeed, if it is not, we can replace the section *I* with another symmetric section centered at the given Weiestrass point; then by Remarks 4.3 and 4.4 (applied to $f=f_p$) this new section yields a special flow which also satisfies the assumption of Theorem 1.2 and both special flows have the same spectral properties since they are both metrically isomorphic to the same surface flow.)

We now claim that the sequence of good rigidity towers given by Lemma 4.10 can be choosen so that19Indeed, since the midpoint $x_0$ of *I* belongs to the core curve of $C_n$ for every *n*, we can choose $J_n=[{a}_n,{b}_n]\subset I$ such that $({a}_n-\varepsilon _n,{b}_n+\varepsilon _n)$ is the unique connected component of the intersection *I* with $C_n$ that contains $x_0$ (see the proof of Lemma 4.10). Then $x_0=({a}_n + {b}_n)/2$. Fix $0<c<1/4$ so that, for every *n* sufficiently large, $\varepsilon _n\le c/q_n$. Then, $x_0\in [a_n+2c/q_n,b_n-2c/q_n] \subset [a_n,b_n]$. Consider now $T^{-h_n}x_0$. Since $|T^{-h_n}x_0-x_0|\le \varepsilon _n\le c/q_n$ and $x_0\in [a_n+2c/q_n,b_n-2c/q_n]$, we have that also $T^{-h_n}x_0\in [a_n+c/q_n,b_n-c/q_n]$, which concludes the proof of (19).

From (19), since $x_0, T^{-h_n}x_0\in [a_n+c/q_n,b_n-c/q_n]\subset (a_n,b_n)$ which by assumption is a continuity interval for $S_{h_n}(f)$ (see the remark after the Definition 4.1), we deduce by Corollary 4.6 that there exists $x_n\in [a_n+c/q_n,b_n-c/q_n]$ such that $S_{h_n}(f')(x_n)=0$.

This shows that all assumptions of Proposition 4.7 hold for the special flow $(T^f_t)_{t \in \mathbb {R}}$ over $T\in \mathscr {F}_{\pi }$ and hence (by Proposition 4.7), that $(T^f_t)_{t \in \mathbb {R}}$ has purely singular spectrum.

$$\square $$

#### Proof of Theorem 1.1

Any $(\varphi _t)_{t\in \mathbb {R}}$ locally Hamiltonian flow with two simple isomorphic saddles on *M* of genus two, by Corollary 2.2, is metrically isomorphic to a special flow over *T* with the symmetric permutation $\pi$ (given by $\pi (i)=4-i$, $0\le i <5$) under $f\in \mathcal {S}\text {ym}\mathcal {L}\text {og} \left( \sqcup _{i=0}^{4}I_i \right)$ (and hence has the same ergodic and spectral properties). By Theorem 1.2, for almost every choice of the lengths $\beta _{i+1}-\beta _i$ of *T*, such special flow has purely singular spectrum. This, by Remark 2.3, implies singularity of the spectrum for a full measure set of locally Hamiltonian flows in the isomorphic saddles locus $\mathcal {K}$ with respect to the Katok fundamental class (defined in Sect. 2.4). $\quad \square$

## Translation Surfaces Well Approximated by Single Cylinders

In this section we will prove the results on the abundance of single cylinders in translation surfaces stated in Sect. 1.3, namely Theorem 1.4 and Proposition 1.3. Let us first show how Proposition 1.3 follows from Theorem 1.4.

### Proof of Proposition 1.3

Let $\psi (t)=\frac{1}{t^2\log t}$. Then $\psi$ satisfies the assumptions of Theorem 1.4. Moreover let $\epsilon _n=\frac{1}{n}$. Notice that a.e. $(M,\omega )$ belongs to the intersection of the full measure sets coming from Theorem 1.4 (intersection over the $(\epsilon _n)_{n\in \mathbb {N}}$). It remains to notice that every such $(M,\omega )$ satisfies the assertion of Proposition 1.3. $\quad \square$

The rest of this section is devoted to the proof of Theorem 1.4. From now on we will constantly assume that $0<\epsilon <1/2$. For every $\theta \in S^1$ and $r>0$ let $B(\theta ,r)=\{\phi \in S^1:\Vert \phi -\theta \Vert <r\}$. Let $\mathcal {C}$ be a connected component in the moduli space of area one translation surfaces. Theorem 1.4 will be a consequence of the following result.

### Proposition 5.1

Let $\psi :\mathbb {R}^+ \rightarrow \mathbb {R}^+$ be non-increasing so that $t\psi (t)\le 1$ for *t* large enough and $\int _1^{+\infty } t \psi (t)=\infty$. For every $0<\epsilon <1/2$ there exists $0<c\le 1$ such that a.e. $(M,\omega )\in \mathcal {C}$ and every interval $J\subset S^1$ satisfies

20 $$\begin{aligned} cT^2 \lambda (J)<\#\{C\in {\mathcal {C}yl}^\epsilon _\omega :\ell (C)\le T\text { and }\theta _C \in J\} \text { for all }T\ge T_{\omega ,J}, \end{aligned}$$

for some $T_{\omega ,J}>0$. Moreover, if (20) holds, then for $T\ge \max (T_{\omega , J}, 36/(c\lambda (J)))$, we have

$$\begin{aligned}\lambda \Big (\bigcup _{\{C\in {\mathcal {C}yl}^\epsilon _\omega :\ell (C)\ge T\}}B(\theta _C, \psi (\ell (C)))\cap J\Big )\ge \frac{c}{9}\lambda (J).\end{aligned}$$

The above result can be proved by a modification of the methods of [Ch] and [MaTrWe]. We will present a full proof for completeness in Sect. 5.1.

Let us now show how it implies Theorem 1.4.

### Proof of Theorem 1.4

Let

$$\begin{aligned}W^\psi _{\omega ,m}:=\bigcup _{\{C\in {\mathcal {C}yl}^\epsilon _\omega :\ell (C)\ge m\}}B(\theta _C,\psi (\ell (C))).\end{aligned}$$

Then the sequence of sets $(W^\psi _{\omega ,m})_{m\ge 1}$ is non-increasing with $\bigcap _{m\ge 1}W^\psi _{\omega ,m}=W^\psi _{\omega }$. To prove (1) we need to show that for a.e. $(M,\omega )\in \mathcal {C}$ and every $m\ge 1$ the set $W^\psi _{\omega ,m}\subset S^1$ has full measure.

In view of Proposition 5.1, for a.e. $(M,\omega )\in \mathcal {C}$ and any interval $J\subset S^1$ we have

21 $$\begin{aligned} \frac{\lambda \big (W^\psi _{\omega ,m}\cap J\big )}{\lambda (J)}\ge \frac{c}{9}\text { if }m\ge T_{\omega ,J}\text { and }{m\ge 36/(c\lambda (J))}. \end{aligned}$$

Take any translation surface $(M,\omega )$ satisfying the above condition and suppose, contrary to our claim, that for some $m_0\ge 1$ the set $W^\psi _{\omega ,m_0}\subset S^1$ does not have full measure. By the Lebesgue density theorem there exists an interval $J\subset S^1$ such that

$$\begin{aligned}\frac{\lambda \big (W^\psi _{\omega ,m}\cap J\big )}{\lambda (J)} \le \frac{\lambda \big (W^\psi _{\omega ,m_0}\cap J\big )}{\lambda (J)}<\frac{c}{9} \text { for all }m\ge m_0,\end{aligned}$$

contrary to (21). This gives (1).

Denote by $A\subset \mathcal {C}$ the set of translation sufaces $(M,\omega )\in \mathcal {C}$ for which there exists a sequence $(C_i)_{i\ge 1}$ in ${\mathcal {C}yl}_\omega ^\epsilon$ such that $\ell (C_i)\rightarrow +\infty$ as $i\rightarrow +\infty$ and $\Vert \theta _{C_i}-\frac{\pi }{2}\Vert <\psi (\ell (C_i))$ for all $i\ge 1$. In view of (1) there exists a subset $A'\subset \mathcal {C}$ with $\nu _C(A')=1$ such that if $(M,\omega )\in A'$ then for every $\phi \in W^{\psi }_\omega$ we have

$$\begin{aligned}r_{\pi /2-\phi }\omega \in A\quad \text {and}\quad \lambda (W^{\psi }_\omega )=1.\end{aligned}$$

Let us consider the continuous map

$$\begin{aligned}\Delta :S^1\times \mathcal {C}\rightarrow \mathcal {C},\quad \Delta (\theta ,\omega )=r_{\pi /2-\theta }\omega .\end{aligned}$$

By Fubini’s theorem and the invariance of $\nu _{{\mathcal {C}}}$ under the action of rotations $(r_\theta )_{\theta \in S^1}$, we have $\Delta _*(\lambda \times \nu _{{\mathcal {C}}})=\nu _{{\mathcal {C}}}$. Moreover,

$$\begin{aligned}\bigcup _{\omega \in A'}(W_{\omega }^\psi \times \{\omega \})\subset \Delta ^{-1}(A).\end{aligned}$$

Using again Fubini’s theorem, we obtain

$$\begin{aligned}1=(\lambda \times \nu _{{\mathcal {C}}})(\Delta ^{-1}(A))=\nu _{{\mathcal {C}}}(A),\end{aligned}$$

which completes the proof. $\quad \square$

### Proof of Proposition 5.1

The first part of Proposition 5.1 (i.e. (20)) is an immediate consequence of the following result, which follows by Theorem 1.9 in [Vor]:

#### Theorem 5.2

For a.e. translation surface $(M,\omega )\in \mathcal {C}$ and all intervals $J\subset S^1$, $I\subset [0,1]$ we have

$$\begin{aligned}\lim _{T\rightarrow +\infty }\frac{\#\{C\in {\mathcal {C}yl}_\omega : \ell (C) \le T,\theta _C\in J,a(C)\in I\}}{T^2}=c_1(\mathcal {C})\lambda (J)|I|^{m_{\mathcal {C}}-1}.\end{aligned}$$

So it remains to prove the second part of Proposition 5.1. For this we first state some additional lemmas.

#### Lemma 5.3

Let $(M,\omega )$ be any translation surface. If $x\in M$ belongs to two different cylinders $C, C'\in {\mathcal {C}yl}_\omega$ then $\Vert \theta _{C}-\theta _{C'}\Vert \ge \frac{\max \{a(C),a(C')\}}{\ell (C)\ell (C')}$.

#### Proof

For every $\theta \in S^1$ denote by $(h^\theta _t)_{t\in \mathbb {R}}$ the directional translation flow on $(M,\omega )$ in direction $\theta$. Notice that the width of the cylinder *C* is $\frac{a(C)}{\ell (C)}$. Suppose that $x\in C$ is a periodic point for $(h^\phi _t)_{t\in \mathbb {R}}$ for some $\phi \ne \theta _C$ and $R>0$ is its minimal period, i.e. $h_s^{\phi }(x)=h_{s-R}^{\phi }(x)$ for all $s\in \mathbb {R}$. Choose $s\in \mathbb {R}$ so that $h_s^{\phi }(x)$ is just leaving the periodic cylinder *C*. So $h^{\phi }_{s-t}(x)\in C$ for all $0<t<\frac{a(C)}{\ell (C)}|\csc (\theta _C-\phi )|$ and in particular $h_{s-t}^{\phi }(x)\ne h^{\phi }_s(x)$. Therefore $R\ge \frac{a(C)}{\ell (C)}|\csc (\theta _C-\phi )|$ which implies that $\Vert \theta _C-\phi \Vert \ge \frac{a(C)}{R\ell (C)}$. $\quad \square$

#### Corollary 5.4

If $0<\epsilon <1/2$ then the set

$$\begin{aligned}\{\theta _C\in S^1:C \in {\mathcal {C}yl}^\epsilon _\omega \text { such that }\ell (C)\le T\}\end{aligned}$$

is $\frac{1-\epsilon }{T^2}$ separated. In particular, for any interval *J* and $T>0$ we have

$$\begin{aligned}\#\{C\in {\mathcal {C}yl}^\epsilon _\omega :\ell (C)\le T\text { and }\theta _C \in J\}\le 2T^2 \lambda (J)+1.\end{aligned}$$

#### Proof

Since the cylinders have area greater than $\frac{1}{2}$, any pair of cylinders must share a point. The statement then follows from Lemma 5.3. $\quad \square$

For any interval $J=B(\theta ,r)\subset S^1$ and any $s>0$ let $J^{+s}:=B(\theta ,r+s)$.

#### Lemma 5.5

Let $\sigma :=18\sqrt{c^{-1}}>1$ and $0<\epsilon < 1/2$. Assume that $J\subset S^1$ is an interval and $T\ge 36/(c\lambda (J))$ satisfy (20) and

22 $$\begin{aligned} \lambda \Big (\bigcup _{\{C\in {\mathcal {C}yl}^\epsilon _\omega :L\ge \ell (C)\ge T\}}B(\theta _C, \psi (\ell (C)))\cap J\Big )<\frac{c}{9}\lambda (J) \end{aligned}$$

for some $L>T$. Then

$$\begin{aligned} \lambda \Big (\Big (&\bigcup _{\{C\in {\mathcal {C}yl}^\epsilon _\omega : \sigma L\ge \ell (C)\ge L\}}B(\theta _C, \psi (\ell (C))) \setminus \bigcup _{\{C\in {\mathcal {C}yl}^\epsilon _\omega :L \ge \ell (C)\ge T\}}B(\theta _C, \psi (\ell (C)))\Big )\cap J\Big )\\&>\min \{{(\sigma L)^2}\psi (\sigma L),1 \}\frac{c}{4}\lambda (J). \end{aligned}$$

#### Proof

As $\sigma L>L>T\ge T_{\omega ,J}$, by (20) and Corollary 5.4, the set

$$\begin{aligned}\Theta :=J\cap \bigcup _{\{C\in {\mathcal {C}yl}^\epsilon _{\omega }:\ell (C) \le \sigma L\}}\{\theta _C\}\subset S^1\end{aligned}$$

has at least $c(\sigma L)^2 \lambda (J)$ points that are $\frac{1}{2(\sigma L)^2}$ separated. Denote by $\partial \Theta$ the set of two points in $\Theta$ which are the closest to the ends of the interval *J*. Then for every $\theta \in \Theta \setminus \partial \Theta$ we have

23 $$\begin{aligned} B\big (\theta , \min \{\psi (\sigma L),(\sigma L)^{-2}/2\}\big )\subset J. \end{aligned}$$

Since $\psi (T)\le 1/T\le c\lambda (J)/36$, by Corollary 5.4, we have

24 $$\begin{aligned} \#\{C\in {\mathcal {C}yl}^\epsilon _\omega :L\ge \ell (C)\ge T,\;\theta _C \in J^{+\psi (T)}\} \le 2\lambda (J^{+\psi (T)})L^2+1\le 4\lambda (J)L^2+1.\nonumber \\ \end{aligned}$$

Moreover

25 $$\begin{aligned} \bigcup _{\{C\in {\mathcal {C}yl}^\epsilon _\omega :L\ge \ell (C)\ge T\}}B(\theta _C, \psi (\ell (C)))\cap J \subset \bigcup _{\{C\in {\mathcal {C}yl}^\epsilon _\omega :L\ge \ell (C)\ge T,\;\theta _C \in J^{+\psi (T)}\}}B(\theta _C,\psi (\ell (C))) \end{aligned}$$

and

26 $$\begin{aligned} \begin{aligned} \lambda&\Big (\bigcup _{\{C\in {\mathcal {C}yl}^\epsilon _\omega :L\ge \ell (C)\ge T,\;\theta _C \in J^{+\psi (T)}\}}B(\theta _C,\psi (\ell (C)))\Big )\\&\le \lambda \Big (\bigcup _{\{C\in {\mathcal {C}yl}^\epsilon _\omega :L\ge \ell (C)\ge T\}}B(\theta _C, \psi (\ell (C)))\cap J\Big )+4\psi (T)<\frac{2c}{9}\lambda (J). \end{aligned} \end{aligned}$$

In view of (26) and (24), the cardinality of the set $\Theta ^*\subset \Theta$ of points $\theta \in \Theta$ such that

$$\begin{aligned}B\big (\theta , \min \{\psi (\sigma L),(\sigma L)^{-2}/2\}\big )\cap \bigcup _{\{C\in {\mathcal {C}yl}^\epsilon _\omega :L\ge \ell (C)\ge T,\;\theta _C \in J^{+\psi (T)}\}} B(\theta _C,\psi (\ell (C)))\ne \emptyset \end{aligned}$$

is at most

$$\begin{aligned} 3(4L^2\lambda (J)+1)+\frac{4c}{9} \lambda (J) {(\sigma L)^2}. \end{aligned}$$

Indeed, the union of *N* intervals with total measure $\mu$ meets at most $\mu /\epsilon + N$ points which are $\epsilon$-separated. As elements of $\Theta$ are $1/(2(\sigma L)^2)$-separated, it follows that there are at most

$$\begin{aligned}\frac{\frac{2c}{9}\lambda (J)}{\frac{1}{2(\sigma L)^2}} +4L^2\lambda (J)+1=\frac{4c}{9} \lambda (J) {(\sigma L)^2}+4L^2\lambda (J)+1\end{aligned}$$

elements of $\Theta ^*$ such that

27 $$\begin{aligned} \theta \in \bigcup _{\{C\in {\mathcal {C}yl}^\epsilon _\omega :L\ge \ell (C)\ge T,\;\theta _C \in J^{+\psi (T)}\}} B(\theta _C,\psi (\ell (C))). \end{aligned}$$

Suppose that $\theta \in \Theta ^*$ does not meet (27). Then $\theta$ is in the $1/(2(\sigma L)^2)$-neighbourhood of an interval $B(\theta _C,\psi (\ell (C)))$ but it does not belong to $B(\theta _C,\psi (\ell (C)))$. As elements of $\Theta$ are $1/(2(\sigma L)^2)$-separated, for every *C* there are at most two elements of $\Theta ^*$ which do not meet (27). Thus

$$\begin{aligned} \#\Theta ^*\le & {} \frac{4c}{9} \lambda (J) {(\sigma L)^2}+4L^2\lambda (J)+1+2(4L^2\lambda (J)+1)\\&=3(4L^2\lambda (J)+1)+\frac{4c}{9} \lambda (J) {(\sigma L)^2}. \end{aligned}$$

Since $\#\Theta \ge c(\sigma L)^2 \lambda (J)$, $\sigma =18/\sqrt{c}$ and $L>T\ge 36/(c\lambda (J))$, this gives

28 $$\begin{aligned} \begin{aligned} \#(\Theta \setminus (\partial \Theta \cup \Theta ^*))&\ge c(\sigma L)^2\lambda (J)- 12L^2\lambda (J)-5-\frac{4c}{9} \lambda (J) (\sigma L)^2\\&\ge c\frac{5}{9} (\sigma L)^2 \lambda (J)-17L^2\lambda (J)>\frac{1}{2} c( \sigma L)^2 \lambda (J). \end{aligned} \end{aligned}$$

As $\psi$ is non-increasing, by (23) and the definition of $\Theta ^*$, for every $\theta \in \Theta \setminus (\partial \Theta \cup \Theta ^*)$ we have $B(\theta , \min \{\psi (\sigma L),(\sigma L)^{-2}/2\})$ is a subset of

$$\begin{aligned}&\Big (\!\bigcup _{\{C\in {\mathcal {C}yl}^\epsilon _\omega :\sigma L \ge \ell (C)\ge L\}}B(\theta _C, \psi (\ell (C)))\!\setminus \!\bigcup _{\{C\in {\mathcal {C}yl}^\epsilon _\omega :L\ge \ell (C) \ge T,\theta _C\in J^{+\psi (T)}\}}B(\theta _C, \psi (\ell (C)))\!\Big )\cap J\\&\qquad \subset \Big (\bigcup _{\{C\in {\mathcal {C}yl}^\epsilon _\omega :\sigma L\ge \ell (C)\ge L\}}B(\theta _C, \psi (\ell (C)))\setminus \bigcup _{\{C\in {\mathcal {C}yl}^\epsilon _\omega :L\ge \ell (C)\ge T\}}B(\theta _C, \psi (\ell (C)))\Big )\cap J, \end{aligned}$$

where the last inclusion follows from (25). Since the centers of intervals are $(\sigma L)^{-2}/2$ separated, by (28), the measure of the last set is at least

$$\begin{aligned}\frac{1}{4} c( \sigma L)^2 \lambda (J)\min \{\psi (\sigma L),(\sigma L)^{-2}\},\end{aligned}$$

which completes the proof. $\quad \square$

#### Lemma 5.6

If $\psi :\mathbb {R}^+\rightarrow \mathbb {R}^+$ is bounded, non-increasing and $\int _{1}^\infty t\psi (t)=+\infty$ then for any $\sigma >1$ we have

$$\begin{aligned}\sum _{k=0}^\infty \sigma ^{2k}\psi (\sigma ^k)=+\infty .\end{aligned}$$

#### Proof

Lemma follows directly from the following

$$\begin{aligned} \int _{1}^\infty t\psi (t)dt=&\sum _{k=0}^\infty \int _{\sigma ^k}^{\sigma ^{k+1}}t\psi (t)dt\le \sum _{k=0}^\infty (\sigma ^{k+1}-\sigma ^k)\sigma ^{k+1}\psi (\sigma ^k)\\ =&\sum _{k=0}^\infty \sigma (\sigma -1)\sigma ^{2k}\psi (\sigma ^k). \end{aligned}$$

$$\square $$

#### Proof of Proposition 5.1

Recall that we only need to show the second part (we already know that (20) holds).

To prove the result we need to show that for every interval $J\subset S^1$ there exists $k\ge \log _\sigma T$ such that

29 $$\begin{aligned} \lambda \Big (\bigcup _{\{C\in {\mathcal {C}yl}^\epsilon _\omega :\sigma ^k\ge \ell (C)\ge T\}}B(\theta _C, \psi (\ell (C)))\cap J\Big )\ge \frac{c}{9}\lambda (J). \end{aligned}$$

Suppose, contrary to our claim, that for all $k\ge \log _\sigma T$ (29) does not hold. By Lemmas 5.5 and 5.6, we have

$$\begin{aligned}&\lambda \Big (\bigcup _{\{C\in {\mathcal {C}yl}^\epsilon _\omega :\ell (C)\ge T\}} B(\theta _C, \psi (\ell (C)))\cap J\Big )\\&\quad \ge \sum _{k\ge \log _{\sigma }(T)} \lambda \Big (\Big (\bigcup _{\{C\in {\mathcal {C}yl}^\epsilon _\omega :\sigma ^{k+1} \ge \ell (C)\ge \sigma ^k\}}B(\theta _C, \psi (\ell (C))) \setminus \bigcup _{\{C\in {\mathcal {C}yl}^\epsilon _\omega :\sigma ^k\ge \ell (C)\ge T\}}B(\theta _C, \psi (\ell (C)))\Big )\cap J\Big )\\&\quad \ge \sum _{k\ge \log _{\sigma }(T)}\min \{{\sigma ^{2k}}\psi (\sigma ^k),1 \}\frac{c}{4}\lambda (J)=+\infty , \end{aligned}$$

which is a contradiction. $\quad \square$

## Footnotes

1 Locally Hamiltonian flows (when minimal, or restricted to a minimal component) can indeed be seen as *singular time-reparametrizations* of translation flows (see Remark 2.1) and properties such as ergodicity and minimality depend only on the flow orbits and not on the time-reparametrization.

2 The result on the singular nature of the spectrum proved in this paper was furthermore explicitly suggested by A. Katok to A. Kanigowski in private communication.

3 The authors in [FL03] show that special flows over rotations under a symmetric logarithm, for a full measure set of frequencies, are *spectrally disjoint* from all mixing flows (see Theorem 12, from which it follows in particular that the spectrum is purely singular).

4 We usually assume that $|I|\le 1$.

5 Saddle loops homologous to zero are indeed persistent under small perturbations, see §2.1 in [Zor] or Lemma 2.4 in [Rav].

6 *Minimal components* are subsurfaces (possibly with boundary) on which the (restriction of the) flow is *minimal*, in the sense that all semi-infinite trajectories are dense. As proved independently by Maier [Mai], Levitt [Lev] and Zorich [Zor]), each smooth area-preserving flow can be decomposed into up to *g* minimal components and periodic components, i.e. subsurfaces (possibly with boundary) on which all orbits are closed and periodic (as the disk filled by periodic orbits in Fig. 3a).

7 Symmetry, or asymmetry, of the logarithmic singularities are crucial in determining the mixing properties of the flow, see Sect. 2.8. Asymmetry is usually introduced by the presence of saddle loops homologous to zero, we refer for example [Rav] for details.
